# Releasing the brakes of tumor immunity with anti-PD-L1 and pushing its accelerator with L19–IL2 cures poorly immunogenic tumors when combined with radiotherapy

**DOI:** 10.1136/jitc-2020-001764

**Published:** 2021-03-09

**Authors:** Veronica Olivo Pimentel, Damiënne Marcus, Alexander MA van der Wiel, Natasja G Lieuwes, Rianne Biemans, Relinde IY Lieverse, Dario Neri, Jan Theys, Ala Yaromina, Ludwig J Dubois, Philippe Lambin

**Affiliations:** 1 The M-Lab, Department of Precision Medicine, GROW - School for Oncology, Maastricht University, Maastricht, The Netherlands; 2 Department of Chemistry and Applied Biosciences, Swiss Federal Institute of Technology, Zurich, Switzerland

**Keywords:** radiotherapy, tumor microenvironment, immunotherapy

## Abstract

**Background:**

Poorly immunogenic tumors are hardly responsive to immunotherapies such as immune checkpoint blockade (ICB) and are, therefore, a therapeutic challenge. Combination with other immunotherapies and/or immunogenic therapies, such as radiotherapy (RT), could make these tumors more immune responsive. We have previously shown that the immunocytokine L19–IL2 combined with single-dose RT resulted in 75% tumor remission and a 20% curative abscopal effect in the T cell-inflamed C51 colon carcinoma model. This treatment schedule was associated with the upregulation of inhibitory immune checkpoint (IC) molecules on tumor-infiltrating T cells, leading to only tumor growth delay in the poorly immunogenic Lewis lung carcinoma (LLC) model.

**Methods:**

We aimed to trigger curative therapeutic responses in three tumor models (LLC, C51 and CT26) by “pushing the accelerator” of tumor immunity with L19–IL2 and/or “releasing the brakes” with ICB, such as antibodies directed against cytotoxic T lymphocyte associated protein 4 (CTLA-4), programmed death 1 (PD-1) or its ligand (PD-L1), combined with single-dose RT (10 Gy or 5 Gy). Primary tumor endpoint was defined as time to reach four times the size of tumor volume at start of treatment (4T×SV). Multivariate analysis of 4T×SV was performed using the Cox proportional hazards model comparing each treatment group with controls. Causal involvement of T and natural killer (NK) cells in the anti-tumor effect was assessed by in vivo depletion of T, NK or both cell populations. Immune profiling was performed using flow cytometry on single cell suspensions from spleens, bone marrow, tumors and blood.

**Results:**

Combining RT, anti-PD-L1 and L19–IL2 cured 38% of LLC tumors, which was both CD8^+^ T and NK cell dependent. LLC tumors were resistant to RT +anti-PD-L1 likely explained by the upregulation of other IC molecules and increased T regulatory cell tumor infiltration. RT+L19–IL2 outperformed RT+ICB in C51 tumors; effects were comparable in CT26 tumors. Triple combinations were not superior to RT+L19–IL2 in both these models.

**Conclusions:**

This study demonstrated that combinatorial strategies rationally designed on biological effects can turn immunotherapy-resistant tumors into immunologically responsive tumors. This hypothesis is currently being tested in the international multicentric randomized phase 2 trial: ImmunoSABR (NCT03705403).

## Introduction

Despite the emerging success of immune checkpoint blockade (ICB), curing advanced cancer in patients with otherwise poor prognosis, response rates to ICB are still low,^1^ mainly due to primary resistance of poorly immunogenic, lymphocyte-excluded cold tumors,^2^ as opposed to lymphocyte-infiltrated hot tumors susceptible to ICB.^3^ Inhibitory immune checkpoint (IC) molecules normally regulate immune responses to avoid excessive inflammation and autoimmunity.^4^ After chronic antigen stimulation, as it occurs in cancer, tumor-specific T cells upregulate IC expression, whose engagement leads to T cell exhaustion, thus impairing the anti-tumor immune response. Besides, tumor cells upregulate IC molecule expression as an acquired mechanism to escape immunity. ICB circumvents the immunosuppressive effects of these inhibitory molecules by reinvigorating the functions of pre-existing exhausted tumor-infiltrating lymphocytes (TILs) and/or preventing the exhaustion of treatment-induced de novo TILs.^5^ ICB has quickly gained approval to treat various metastatic cancer types,^6^ changing cancer treatment practice over the last decade.

Low response rates to ICB monotherapy can be overcome by combination with other immunotherapies with independent mechanisms of action, such as immunostimulating cytokines or agonistic antibodies. Another strategy is by combination with immunogenic genotoxic therapies, such as radiotherapy (RT). RT is one of the most widely used treatment options for solid tumors, with half of newly diagnosed patients receiving RT during their disease.^7^ RT-induced damage results in tumor-immune recognition of cancer cells, dendritic cell maturation, and cross-priming of naïve CD8^+^ T cells, leading to their differentiation into tumor-specific T cells.^8^ In addition, RT increases the expression of cellular ligands of activating natural killer (NK) receptors on tumor cells, increasing their anti-tumor cytotoxic activity.^9^ Although RT by itself is often not sufficient to mount an effective anti-tumor immune response that will result in tumor clearance, its immunogenicity can be efficiently increased with immunotherapies. Synergy between RT and systemic administration of (immuno-)cytokines against cancer has been evidenced.^10–16^ L19–IL2 is an immunocytokine consisting of a fusion protein of the variable fraction of a human antibody (L19) directed against the extra-domain B (ED-B) of an embryonic splice variant of fibronectin expressed in the tumor neovasculature and human recombinant interleukin-2 (IL2).^17^ Thus, the efficacy of L19–IL2 highly depends on the expression of ED-B in the tumor.^18^ We have previously shown that the addition of the tumor-targeted antibody-based immunocytokine L19–IL2 to single-dose RT (10 Gy) resulted in 75% primary tumor remission, a 20% curative abscopal effect, and a protective anti-tumor immune memory response in a C51 murine colon carcinoma model.^19 20^ We also demonstrated an additive effect between a single-dose 10 Gy and L19–IL2 in the NK cell-sensitive F9 teratocarcinoma mouse model.^21^ In the Lewis lung carcinoma (LLC), which has been proven to be a poorly immunogenic tumor model,^22 23^ only a significant tumor growth delay was observed, associated with the upregulation of IC molecules on tumor-infiltrating CD8^+^ T cells.^19^ Likewise, the anti-tumor synergistic effect of RT and ICB has been extensively proven both pre-clinically and clinically in hot tumors.^24^ However, less is known about effective combination strategies yielding durable responses in poorly immunogenic tumors. Since we have observed that RT combined with L19–IL2 upregulates expression of IC molecules, we reasoned that exploiting the interaction between RT, L19–IL2, and ICB has high potential to achieve tumor cure. RT triggers the cross-priming of naïve CD8^+^ T cells allowing their differentiation into cytotoxic CD8^+^ T cells, which proliferate in the presence of L19–IL2 at the tumor site. We, thereby, hypothesize that “releasing the brakes” of the immune system with ICB and “pushing its accelerator” with L19–IL2 boosts anti-tumor immune responses when combined with RT. Besides, we evaluate if RT synergizes better with L19–IL2 than ICB. We assessed our hypotheses in three ED-B-positive models: the poorly immunogenic LLC tumor model and the T cell inflamed C51 and CT26 colon carcinoma models.^20 22 23^ Additionally, to understand the immune mechanism(s) of response and resistance to treatment, we investigated the tumor infiltration and status of different immune cell subsets during treatment.

## Material and methods

### Tumor cell lines, reagents and antibodies for in vivo experiments

The LLC cell line (provided by G Molema, UMCG, the Netherlands) syngeneic to C57bl/6 mice, as well as the C51 and CT26 mouse colon carcinoma cell lines (Philogen S.p.A.) syngeneic to Balb/c mice, were cultured in Dulbecco’s Modified Eagle Medium (DMEM; Lonza), supplemented with 10% fetal bovine serum (FBS) in a humidified 5% CO_2_ chamber at 37°C. L19–IL2 (Philogen S.p.A.) was diluted in sterile phosphate-buffered saline (PBS) to 200 µg/mL. For in vivo IC inhibition, rat antibodies directed against cytotoxic T lymphocyte associated protein 4 (anti-CTLA-4, 9D9), programmed death 1 (anti-PD-1, RMP1-14) or its ligand (anti-PD-L1, 10F.9G2) and rat IgG2b control (LFT-2) were diluted in sterile PBS to 2.5 mg/mL (BioXcell). For in vivo cell depletion, rat anti-CD8α (YTS 169.4), rat anti-NK1.1 (PK136), and rat IgG2b control (LTF-2) were diluted in sterile PBS to 2.5 mg/mL (BioXcell).

### Mice and in vivo experiments

All experiments were performed in accordance with local institutional guidelines for animal welfare and were approved by the Animal Ethical Committee of the University of Maastricht, the Netherlands, and were in accordance with the Declaration of Helsinki of 1975 as revised in 2000. In all in vivo experiments, tumors were measured using a Vernier caliper until primary tumor endpoint, defined as the time to reach four times the size of tumor volume at start of treatment (T4×SV). Tumor volume was calculated using the formula (π/6)×length×width×height, each dimension corrected for the skin thickness (0.5 mm). None of the animals were excluded from the study for reasons other than reaching endpoint or completion of the follow-up time. All irradiations were performed using Varian Truebeam linear accelerator (15 MeV) electrons, as previously described.^19 20^

#### Tumor growth delay studies

**Figure 1:**
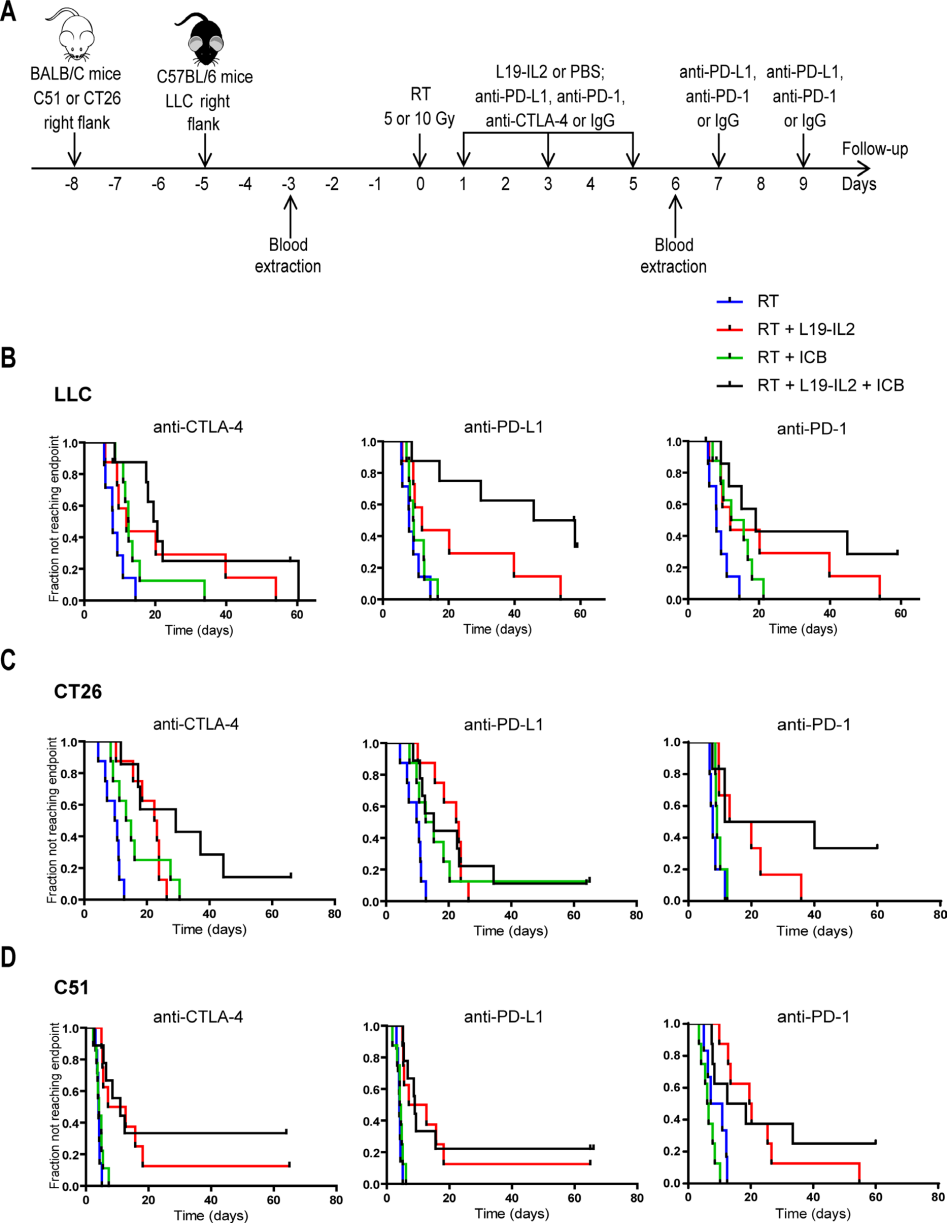
Therapeutic effect of RT combined with L19–IL2 and/or ICB treatment. (A) Treatment schedule. Each mouse was injected with tumor cells on the right flank on day 8 (C51 and CT26 models) or day 5 (LLC model). Blood was withdrawn 3 days before and 6 days after treatment start for flow cytometric analysis. Fraction of tumors not reaching four times start tumor volume in LLC (n=7–8 mice per treatment arm) (B), CT26 (n=7–9) (C) and C51 (n=7–9) (D) tumor models. C51 and CT26 tumors were irradiated with a single dose of 5 Gy and LLC tumors with an isoeffective single dose of 10 Gy. Treatments in the LLC and the CT26 models were performed in one single experiment, while in the C51 model two independent experiments were performed (see online supplemental figure 4). Combinations with anti-PD-1 in the C51 (n=6–8) and CT26 (n=5–6) were tested in an independent experiment. Differences between treatment groups for each tumor model are summarized in table 1. Treatments: blue: RT, red: RT + L19-IL2, green: RT + ICB, black: RT + L19-IL2 + ICB. ICB, immune checkpoint blockade; IgG, immunoglobulin G; LLC, Lewis lung carcinoma; PBS, phosphate-buffered saline; RT, radiotherapy.

C57BL/6 or Balb/c mice were injected in the right flank with 1.5×10^6^ LLC, C51 or CT26 colon carcinoma cells respectively. On an average volume of 200 mm^3^, mice were randomized in different treatment groups: RT+PBS/L19–IL2 (1 mg/kg)+IgG or RT+PBS/L19–IL2+anti-PD-L1/anti-PD-1/anti-CTLA-4 (all 10 mg/kg). Tumors were locally irradiated with 10 Gy (LLC) or 5 Gy (C51 and CT26). PBS/L19–IL2, anti-CTLA-4 and IgG were given intravenous on days 1, 3 and 5 after RT (day 0); anti-PD-1, anti-PD-L1 and IgG were given intraperitoneal 1, 3, 5, 7 and 9 days after RT. An overview of the treatment schedules is presented in figure 1A. Median and ranges of tumor treatment start volumes are depicted in online supplemental table 1.

10.1136/jitc-2020-001764.supp1 Supplementary data

10.1136/jitc-2020-001764.supp5 Supplementary data

There was 150 μL of blood collection from the saphenous vein in 50 μL of 10% sodium heparin (5000 IU/mL, Leo Laboratories) before and 6 days after treatment start to assess the expression of PD-1, PD-L1 and CTLA-4 on circulating T cells (see below). Spleen and bone marrow were collected at endpoint (T4×SV or end of follow-up) as reported previously^19 25^ for analysis of memory T cells by flow cytometry.

#### In vivo depletion study

Anti-NK1.1, anti-CD8α, or IgG (all 10 mg/kg) antibodies were injected intraperitoneal in LLC tumor-bearing mice treated with RT+L19–IL2+anti-PD-L1, 3 days before treatment start and then every 3 days until the end of treatment (6 injections in total). Depletion of CD8^+^ T cells and NK cells was monitored by flow cytometry on peripheral blood collected via puncture of the saphenous vein using non-competing antibodies that recognize a different chain (anti-CD8β-BV510 (H35-17.2, BD Biosciences)) or a different epitope (anti-NK1.1-APC (REA1162, Miltenyi Biotec)). An overview of the experimental setup is shown in figure 4A.

#### Immune profiling studies

**Figure 2:**
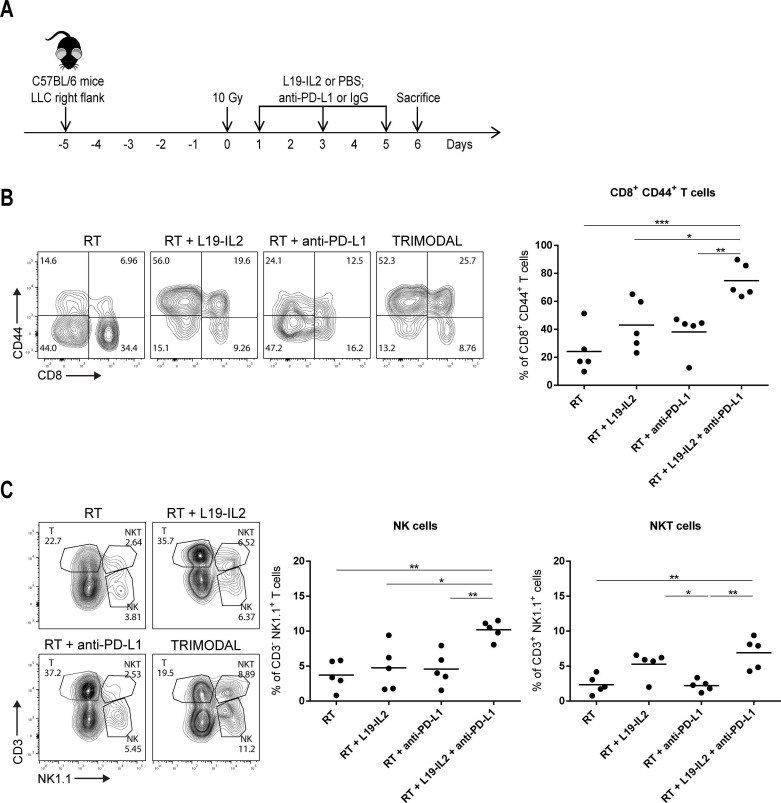
RT+L19–IL2+anti-PD-L1 (trimodal) therapy effect is associated with high infiltration of NK and antigen-experienced CD8^+^ CD44^+^ T cells in the LLC model. (A) Treatment schedule for the evaluation of immunological parameters in the LLC model (n=5 mice per treatment arm), experiment was performed once. Stainings, flow cytometry data acquisition, and analysis of the samples were done in independent duplicates. (B) Representative flow cytometry dot plots of tumor-infiltrating CD8^+^ CD44^+^ (gated on CD3^+^ T cells) T cells 6 days after the start of treatment and quantification. (C) Representative flow cytometry dot plots of tumor-infiltrating T (CD3^+^ NK1.1^-^), NKT (CD3^+^ NK1.1^+^) and NK (CD3^-^ NK1.1^+^) cells and quantifications of NKT and NK cell percentages. IgG, immunoglobulin G; LLC, Lewis lung carcinoma; PBS, phosphate-buffered saline; RT, radiotherapy.

Various immune cell subsets and molecules were investigated at day 6 after treatment onset (experimental setup described in figure 2A). We compared tumor-immune infiltrates of LLC tumors treated with RT, RT+L19–IL2, RT+anti-PD-L1 and RT+L19–IL2+anti-PD-L1.

#### Rechallenge of cured mice with tumor cells

Long-term surviving animals after treatment with single-dose RT+L19–IL2+anti-PD-L1 (LLC: n=3, CT26: n=1 and C51: n=3), single-dose RT+L19–IL2+anti-CTLA-4 (LLC: n=1, CT26: n=1 and C51: n=6), and single-dose RT+L19–IL2+anti-PD-1 (LLC: n=2, CT26: n=2 and C51 n=2) were re-challenged on their right flank with LLC, CT26 or C51 cells (1.5×10^6^ suspended in matrigel): 31±2.1 days (LLC), 55±3.4 days (CT26) and 58±3.5 days (C51) after tumor cure had been observed. These time periods are sufficient to detect all tumor recurrences.^18^ Tumor growth was monitored for 12 days, which is sufficient for tumor growth initiation.

### Flow cytometry

Flow cytometry analysis was performed on freshly isolated blood and frozen-thawed cells from spleens, bone marrow and tumors. Blood was collected via saphenous vein puncture in 10% sodium heparin. Mice were euthanized and sprayed with 70% ethanol. Spleens and tumors were excised and collected in ice-cold Roswell Park Memorial Institute (RPMI) 1640+10% FBS+1% penicillin/streptomycin. Single-cell suspensions of these organs were obtained using a gentleMACS dissociator (Miltenyi Biotec B.V.) and filtered through a 70 µm pore cell strainer (Greiner, Bio-one). Femur and tibia were removed, both ends of bones were carefully sectioned, and bone marrow was flushed using a 26G needle with RPMI 1640+10% FBS+1% penicillin/streptomycin. Red blood cell lysis was performed on single-cell suspensions of spleens, tumors, bone marrow and blood using RBC lysis buffer (eBioscience); blood samples were stained immediately after. Single-cell suspensions were then centrifuged (150 g, 8 min, 4°C) and resuspended in ice-cold freezing medium consisting of 85% FBS, 10% dimethylsulfoxide (DMSO) and 5% of 3 M glucose solution (B. Braun). Then, 1 mL of the suspension was pipetted into cryovials, transferred immediately to −80°C and at least 24 hours later at −196°C in liquid nitrogen. Cells were thawed in a 37°C water bath and diluted in pre-warmed RPMI 1640 medium supplemented with 10% FBS, 1% L-glutamine and 1% penicillin/streptomycin. Cells were spun down (150 g, 8 min at 4°C), resuspended in 2 mL of cold PBS and counted. Then, 2×10^6^ cells were transferred to a V-bottom 96-well transparent plate (Greiner Bio-one) where staining was performed while kept on ice. Cells were stained with LIVE/DEAD Fixable Aqua Dead Cell Stain kit (L/D Aqua, Life Technologies) according to manufacturer’s instructions, washed with fluorescence-activated cell sorting (FACS) buffer (PBS, 2% FBS and 2 mM EDTA), incubated with anti-CD16/32 (2.4G2, BD Biosciences) to block Fc receptors (FcRs) and stained with the following surface marker antibodies for flow cytometry analysis: anti-CD45-PerCP or APC (clone 30F-11), anti-CD3-eFluor450 or FITC (17A2), anti-CD4-APC-H7 (GK1.5), anti-CD8-PerCP (53–6.7), anti-CD44-APC-Cy7 (IM7), anti-CD62L-PE-Cy7 (Mel-14), anti-CD127-PE (SB/199), anti-PD-1-PE-Cy7 (RMP1-30), anti-CD25-APC (PC61) anti-PD-L1-PE (10F.9G2), anti-NK1.1-BV421 (PK136), anti-MHC-II-PE-Cy7 (2G9), anti-CD11c-APC (HL3), anti-CD11b-PE-Cy7 (M1/70), anti-Gr1-efluor450 (RB6-8C5) and anti-Ly6G-APC-Cy7 (1A8) (all from BD Biosciences); anti-F4/80-PerCP (BM8) and anti-Tim3-PE-Cy7 (RMT3-23) (from Biolegend) or anti-CD39-APC-eFluor780 (24DMS1) (eBiosciences). For intracellular staining, cells were washed with FACS buffer, incubated with fixation/permeabilization working solution (eBioscience) according to manufacturer’s instructions, washed with permeabilization buffer (eBioscience) and stained with anti-FoxP3-PE (FJK-16s, eBiosciences) antibody. Fluorescence compensation was defined using single stained cells. Data were acquired using 8-color panels with a FACS Canto II instrument (BD Biosciences) and FacsDIVA V.6.1.2 software (BD Biosciences). Data were analyzed with FlowJo V.10.0.8 (Tree Star) software. Gating strategies are depicted in online supplemental figure 1.

10.1136/jitc-2020-001764.supp2 Supplementary data

### Cytokine and cytotoxic activity of memory CD8^+^ T cells

As previously described,^19^ single-cell suspensions of splenocytes from treated or naïve mice were co-cultured with LLC tumor cells (4:1 effector:target ratio) in the presence of irradiated target cells (single-dose 50 Gy). To assess specific tumor antigen reactivity, splenocytes from treated mice were co-cultured with irradiated (50 Gy) non-specific GL261 glioblastoma cells. After 4 days of co-culture, cells were harvested, washed and re-stimulated overnight with 50 Gy irradiated LLC or GL261 cells in the presence of protein transport inhibitors (Golgiplug and Golgistop, 1 µL/mL, BD Biosciences). For flow cytometry analysis, cells were stained with LIVE/DEAD Fixable Aqua Dead Cell Stain kit, incubated with anti-CD16/32 (2.4G2, BD Biosciences) and then stained with conjugated monoclonal antibodies for cell surface markers anti-CD3-APC (17A2, Biolegend), anti-CD8-PerCP (53–6.7), anti-CD44-APC-Cy7 (IM7), CD127-PE (SB/199) (BD Biosciences) and CD62L-PE-Cy7 (Mel-14, eBiosciences). Cells were then fixed and permeabilized and stained with anti-interferon (IFN) γ-FITC (XMG1.2, Biolegend) and anti-granzyme B-eFluor450 (NGZB, eBiosciences) antibodies.

### Data and statistical analysis

For analysis of the therapeutic effect of the different treatments compared with RT or RT+L19–IL2 as controls, multivariate analysis of time to local failure was performed using the Cox proportional hazards model with the treatment group as a categorical variable. The time of local failure was defined as T4×SV and the results are reported as HRs. The significant HR <1 indicates that combined treatment provides a greater therapeutic effect as compared with RT or RT+L19–IL2. The proportional hazards assumption was tested based on the distribution of Schoenfeld residuals and was not violated for any of the models.

To test whether pretreatment immunological blood parameters changed on treatment, the ratio of the parameter on day 6 after treatment start to the respective pretreatment parameter was compared with day 1 using one sample two-sided t-test. Ratio >1 indicates an increase and ratio <1 indicates a decrease of the parameter. In addition, blood parameters were tested for the association with treatment outcome using univariate Cox proportional hazards model with a blood parameter as a continuous variable.

Statistical analyses were performed using GraphPad Prism Software V.6.03. A one-way ANOVA with Tukey’s post-test was used to determine the statistical differences between the different treatment groups. Flow cytometry data are presented as mean±SD. An unpaired Student t-test with Welch’s correction was used to compare two groups. The log-rank (Mantel-Cox) test was used to compare survival curves in the depletion study. P values smaller than 0.05 were considered as statistically significant: *p≤0.05, **p≤0.01, ***p≤0.001, and ****p≤0.0001.

## Results

### Combination of RT, immunocytokine and IC inhibition causes curative responses in the poorly immunogenic LLC model

We investigated in poorly immunogenic LLC tumors the therapeutic efficacy of an approach combining RT with immunotherapies that further stimulate the immune system (L19–IL2) and prolong the immune response by blocking immunosuppression (ICB). We found that only the addition of anti-PD-L1 to RT+L19–IL2, that is triple combination, yielded a significantly better therapeutic outcome compared to RT+L19–IL2, resulting in 38% tumor cure. This effect was indirectly confirmed in the depletion study (see below). The addition of anti-CTLA-4 and anti-PD-1 did not improve the therapeutic outcome of RT+L19–IL2 (figure 1B, table 1). A similar setup was tested in CT26 and C51 tumor models, applying an isoeffective radiation dose (5 Gy).^20^ Neither the addition of anti-CTLA-4, anti-PD-1 nor anti-PD-L1 to RT+L19–IL2 resulted in improved therapeutic outcomes for both models (figure 1C, D, table 1), which was confirmed in the C51 model in an independent experiment (online supplemental figure 2). Although no significant differences were found between RT+L19–IL2 and trimodal therapies in the CT26 and C51 models, it is to note that, in most instances, trimodal therapy led to one to threefold more tumor remissions than any bimodal combination. Trimodal combination with anti-PD-L1 yielded 11% and 12.5%–22% tumor cure in the CT26 and the C51 models, respectively. RT+L19–IL2+anti-CTLA-4 cured 14% of CT26 and 33%–37.5% of C51 tumors. Finally, trimodal therapy with anti-PD-1 cured 33% of CT26% and 25% of C51 tumors (figure 1, online supplemental figure 2). This is contrasted to 0% and 12.5% tumor cure after RT+L19–IL2 in the CT26 and C51 tumor models, respectively. Individual (re-)growth curves of the different treatment arms are depicted in online supplemental figure 3. Body weight was monitored throughout the experiments to investigate if any treatment combination resulted in systemic toxicity. Overall, no body weight loss higher than 10% was observed, especially in the LLC model, which was fully recoverable (online supplemental figure 4). LLC tumor-bearing mice cured by triple therapy reached tumor cure in 17±2.5 days; cure was reached in 13±3.9 days and 16±2.8 days in CT26 and C51 tumors, respectively. Tumor cure was monitored for 31±2.1 days (LLC), 55±3.4 days (CT26), and 58±3.5 (C51) days. After this follow-up time, cured mice were rechallenged with either LLC, CT26 or C51 tumor cells and tumor formation was monitored for 12 days, since LLC tumors reached an average tumor volume of 200 mm^3^ in 6±0.8 days from the day of tumor challenge in untreated mice, while CT26 and C51 tumors did so in 11±1.0 days; tumor take was 100%, 94%, and 98%, respectively. None of the animals developed tumors after rechallenge during the monitored time, reflective of the development of an immunological memory against tumor cells triggered by triple therapy.

10.1136/jitc-2020-001764.supp3 Supplementary data

10.1136/jitc-2020-001764.supp4 Supplementary data

**Table 1:** Therapeutic effect by RT and immunotherapy combinations. Cox regression analysis comparing all treatments to RT+L19–IL2 or RT monotherapy used as control. HR <1 indicates superior treatment compared with RT+L19–IL2 or RT

Tumor	Comparison of multiple treatments with the control treatment RT +L19–IL2 HR(95% CI) (P value)						
	–	RT+anti-CTLA-4	RT+L19–IL2+anti-CTLA-4	RT+anti-PD-L1	RT+L19–IL2+anti-PD-L1	RT+anti-PD-1	RT+L19–IL2+anti-PD-1
LLC	–	1.71 (0.6 to 4.88) (0.32)	0.49 (0.16 to 1.48) (0.2)	**3.58** **(1.19 to 10.73)** **(0.022)**	**0.28** **(0.08 to 0.92)** **(0.035)**	1.65 (0.57 to 4.72) (0.35)	0.46 (0.14 to 1.47) (0.19)
CT26	–	1.44 (0.53 to 3.9) (0.48)	0.52 (0.17 to 1.57) (0.24)	1.23 (0.43 to 3.49) (0.7)	0.92 (0.33 to 2.52) (0.88)	2.77 (0.72 to 10.62) (0.14)	0.43 (0.1 to 1.78) (0.25)
C51	–	**7.31** **(2.39 to 22.29)** **(<0.0005)**	0.73 (0.24 to 2.18) (0.57)	**9.15** **(2.81 to 29.77)** **(<0.0005)**	0.9 (0.31 to 2.58) (0.84)	9.95 (2.92 to 33.83) (<0.0005)	0.69 (0.23 to 2.02) (0.49)
**Comparison of multiple treatments with the control treatment RT** **HR (95% CI)** **(P value)**							
	**RT+L19–IL2**	**RT+anti-CTLA-4**	**RT+L19–IL2+anti-CTLA-4**	**RT+anti-PD-L1**	**RT+L19–IL2+anti-PD-L1**	**RT+anti-PD-1**	**RT+L19–IL2+anti-PD-1**
LLC	**0.17** **(0.05 to 0.54)** **(0.003)**	**0.29** **(0.1 to 0.84)** **(0.022)**	**0.08** **(0.02 to 0.28)** **(<0.0005)**	0.62 (0.22 to 1.72) (0.35)	**0.05** **(0.01 to 0.18)** **(<0.0005)**	**0.28** **(0.09 to 0.82)** **(0.02)**	**0.08** **(0.02 to 0.28)** **(<0.0005)**
CT26	**0.14** **(0.04 to 0.44)** **(0.001)**	**0.21** **(0.07 to 0.62)** **(0.004)**	**0.07** **(0.02 to 0.26)** **(<0.0005)**	**0.18** **(0.05 to 0.55)** **(0.002)**	**0.13** **(0.04 to 0.41)** **(<0.0005)**	0.48 (0.13 to 1.71) (0.26)	**0.074** **(0.01 to 0.39)** **(0.002)**
C51	**0.07** **(0.01 to 0.25)** **(<0.0005)**	0.51 (0.17 to 1.44) (0.2)	**0.05** **(0.01 to 0.19)** **(<0.0005)**	0.63 (0.22 to 1.8) (0.39)	**0.062** **(0.01 to 0.22)** **(<0.0005)**	1.98 (0.65 to 5.97) (0.2)	**0.14** **(0.03 to 0.52)** **(0.003)**

Significant HR and two-sided p values are shown in bold.

LLC, Lewis lung carcinoma; RT, radiotherapy.

### RT combined with immunocytokines may have a larger therapeutic effect compared with RT combined with ICB

Concurrently, we evaluated whether treatment with the immunocytokine L19–IL2 is superior to ICB (anti-PD-L1, anti-PD-1 or anti-CTLA-4) when combined with RT. The therapeutic outcome of single-dose radiation combined with L19–IL2 or ICB was investigated in the LLC model (table 1). As expected, the addition of L19–IL2 to radiation significantly improved the therapeutic effect, in line with the results of our previous study,^18^ while the enhancement of the RT effect by adding ICB was dependent on the used ICB molecule. Compared with RT, the therapeutic outcome of RT+anti-CTLA-4 and RT+anti-PD-1 was significantly better, while RT+anti-PD-L1 did not improve tumor response. RT combined with L19–IL2 resulted either in significantly better or similar tumor response as compared with RT combined with ICB. Compared with RT+L19–IL2, RT+anti-PD-L1 was therapeutically worse, while RT+anti-CTLA-4 or anti-PD-1 provided similar anti-tumor effects (figure 1B, table 1).

The same treatment schedules were also tested in CT26 and C51 tumors, where the therapeutic effects were dependent on the tumor model (figure 1C, D, table 1). While RT+L19–IL2 resulted in superior tumor response as compared with RT alone in both CT26 and C51 tumor models, which is in agreement with our previously published data,^18^ the therapeutic outcomes of RT+anti-CTLA-4 and RT+anti-PD-L1 as compared with RT were significantly better only in CT26 tumors. The addition of anti-PD-1 to RT did not improve therapeutic outcomes in both models. Taken together, compared with RT+L19–IL2, RT+anti-CTLA-4, anti-PD-L1 or anti-PD-1 outcomes were worse in C51 tumors, while RT+ICBs outcomes were similar to RT+L19–IL2 in CT26 tumors (figure 1C, D, table 1). This data suggests that boosting the immune system with stimulatory molecules such as L19–IL2 may provide a greater therapeutic benefit compared with ICB in some tumors.

### Triple combination therapy increases numbers of CD8^+^ CD44^+^ T cells and NK cells in LLC tumors

Since solely anti-PD-L1 improved the response of LLC tumors to RT+L19–IL2, we performed mechanistic studies to investigate the tumor immune landscape generated after delivering the aforementioned treatments to LLC tumor-bearing mice (figure 2A). We assessed the infiltration of immune cells from both the lymphoid and myeloid line and did not find any major differences in proportions of tumor-infiltrating immune cells from the myeloid line between treatment arms (online supplemental table 2). Furthermore, no differences were observed in CD4^+^ and CD8^+^ T cell proportions, prompting us to investigate if the activation status of T cells differs among the different treatment arms. We, therefore, stained tumor-infiltrating CD8^+^ T cells for CD44, a marker expressed on activated antigen-experienced T cells, and observed CD44 positivity in 74.82%±5.4 of CD8^+^ T cells from tumors treated with triple therapy (RT+L19–IL2+anti-PD-L1). This proportion was higher compared with tumors treated with RT (24.2%±7.3, p<0.001), RT+L19–IL2 (44.7%±10.9, p<0.05) and RT+anti-PD-L1 (37.0%±8.2, p<0.01) (figure 2B). Furthermore, the triple therapy combination increased tumor-infiltration of NK cells (10.2%±0.6) when compared with RT (3.7%±0.9), RT+L19–IL2 (4.7%±1.4) and RT+anti-PD-L1 (4.6%±1.1) (p<0.01 for all comparisons). Also, L19–IL2 increased NKT cell proportions (figure 2C).

10.1136/jitc-2020-001764.supp6 Supplementary data

### Upregulation of checkpoint molecules on peripheral blood T cells is associated with resistance to triple combination therapy

**Figure 3:**
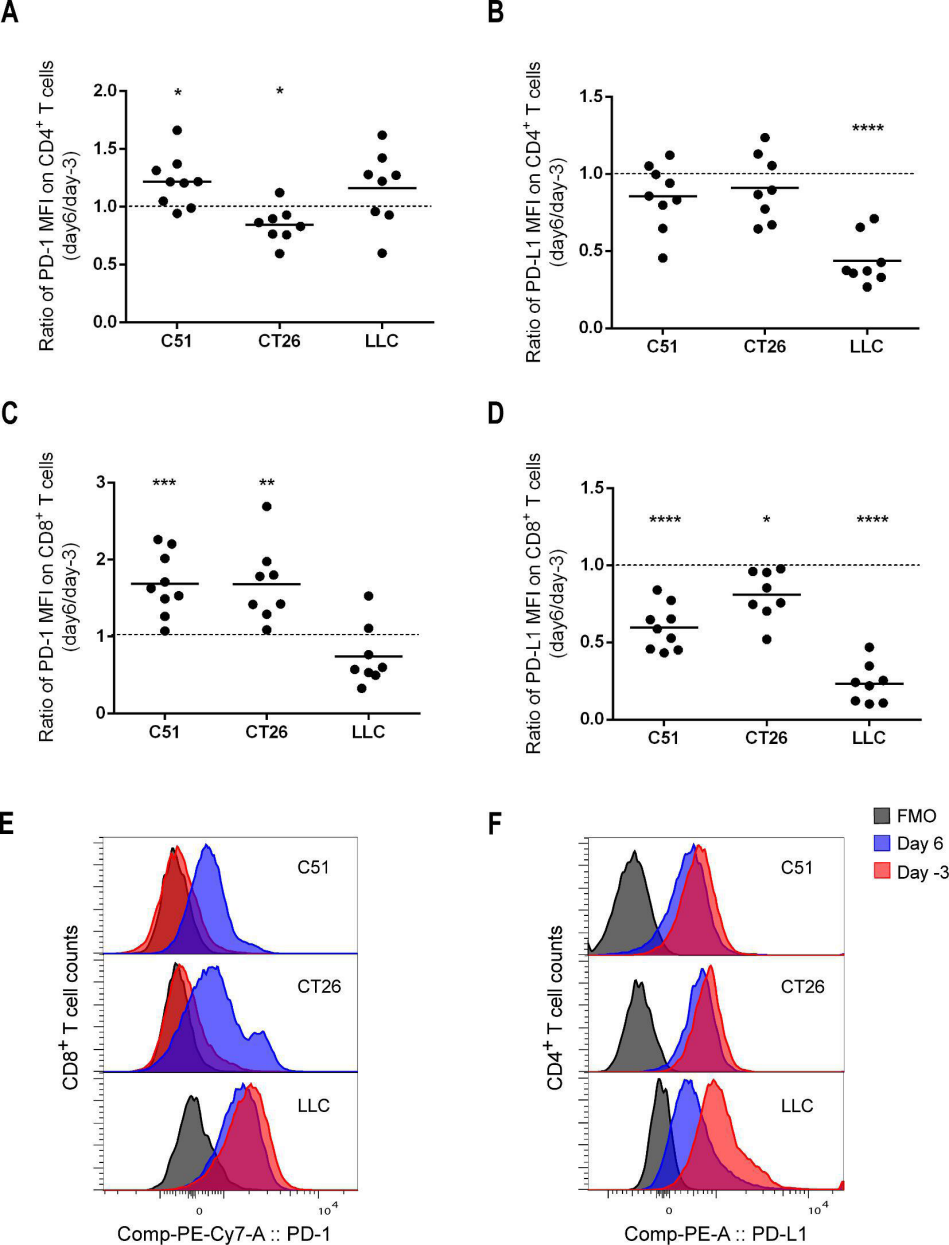
Changes in pretreatment immunological blood parameters (expression of PD-1 and PD-L1 on CD4^+^ and CD8^+^ T cells) during triple therapy (RT+L19–IL2+anti-PD-L1). Blood samples were collected 3 days before treatment (day 3) and on day 6 after treatment start (n=8–9 mice per tumor model, see treatment scheme in figure 1A). Data are reported as the day 6/day -3 ratio of the MFI of PD-L1 on CD4^+^ (A) and CD8^+^ (B) T cells and of PD-1 on CD4^+^ (C) and CD8^+^ T cells (D). Ratio >1 indicates an increase and ratio <1 indicates a decrease in the parameter during therapy. (E) and (F) Representative flow cytometry histograms of (C) and (B), respectively. MFI, median fluorescence intensity.

Based on the results of the analysis of immunological blood parameters, triple therapy using RT+L19–IL2+anti-PD-L1 resulted in a significant increase of PD-1 expression on peripheral CD4^+^ T cells in the C51 tumor model at day 6 after the start of the triple therapy, while this expression decreased in the CT26 tumor model and remained unchanged in the LLC model (figure 3A). While the expression of PD-1 significantly increased on peripheral CD8^+^ T cells in the two non-responsive tumor models C51 and CT26 at day 6 after the start of the triple therapy, the expression of this checkpoint molecule showed a tendency to decrease (p=0.106) in the responsive LLC tumor model (figure 3C, E).

In addition, PD-L1 expression on CD4^+^ T cells significantly (p<0.0001) decreased only in the LLC model and this decrease in CD8^+^ T cells was significantly more pronounced in LLC than in CT26 and C51 models (figure 3B, D, F). It should be noticed that the decrease in the detectable expression of PD-L1 during the triple therapy is expected due to the therapeutic anti-PD-L1 antibody blocking the binding of the fluorescent detection antibody. Therefore, the decrease in PD-L1 detection, which was most pronounced in the responsive LLC tumor model, may serve as an indicator of effective blockade, and thus suppression of the PD-1/PD-L1 signaling pathway. Interestingly, radiation alone resulted in a significant decrease of PD-L1 expression on CD8^+^ and CD4^+^ T cells in the LLC model at day 6 after treatment start, while its expression either remained unchanged on CD8^+^ T cells or significantly increased on CD4^+^ T cells in CT26 and C51 models (online supplemental figure 5A). The addition of L19–IL2 to radiation did not influence the change in PD-L1 expression on T cells in the LLC model, and its increase in CD8^+^ T cells became significant in CT26 and C51 models (online supplemental figure 5B). Taken together, this data suggests that consistent upregulation of IC molecules in response to radiation or combined treatment in the CT26 and C51 tumor models may represent the resistance mechanism to the triple therapy. Furthermore, the decrease in PD-L1 expression on CD4^+^ and CD8^+^ T cells was significantly more pronounced in LLC than in the C51 and CT26 models at day 6 of RT+anti-PD-L1 therapy, suggesting that anti-PD-L1 antibody was less effective in blocking PD-L1 in C51 and CT26 than in LLC model (online supplemental figure 5C). It is relevant to mention that PD-L1 expression on CD8^+^ T cells on different treatments mimics its expression on tumor cells.

10.1136/jitc-2020-001764.supp7 Supplementary data

To test whether immunological blood parameters correlate with tumor response, ie, time to reach endpoint, data of three tumor models per treatment group were fit to univariate Cox proportional hazards model. Pretreatment expression of PD-L1 on peripheral CD8^+^ T cells had a significant positive association with tumor response to triple therapy using RT+L19–IL2+anti-PD-L1 (p=0.044), indicating that subjects with a higher peripheral expression of PD-L1 required a longer time to reach endpoint after triple therapy. PD-L1 expression on peripheral CD8^+^ T cells may serve as a biomarker of tumor response, which can be easily assessed in the clinical situation, but requires further exploration for its prognostic/predictive power in studies testing this novel triple combination. None of the other immunological blood parameters tested (percent CD4^+^, CD8^+^ T cells, expression of PD-1 on CD4^+^, CD8^+^ T cells, expression of PD-L1 on CD4^+^ T cells both pretreatment and at day 6, as well as their change) showed significant association with tumor response to RT+L19–IL2 or RT+L19–IL2+anti-PD-L1 or RT+anti-PD-L1.

### The therapeutic outcome of the triple combination therapy depends on CD8^+^ T and NK cells

**Figure 4:**
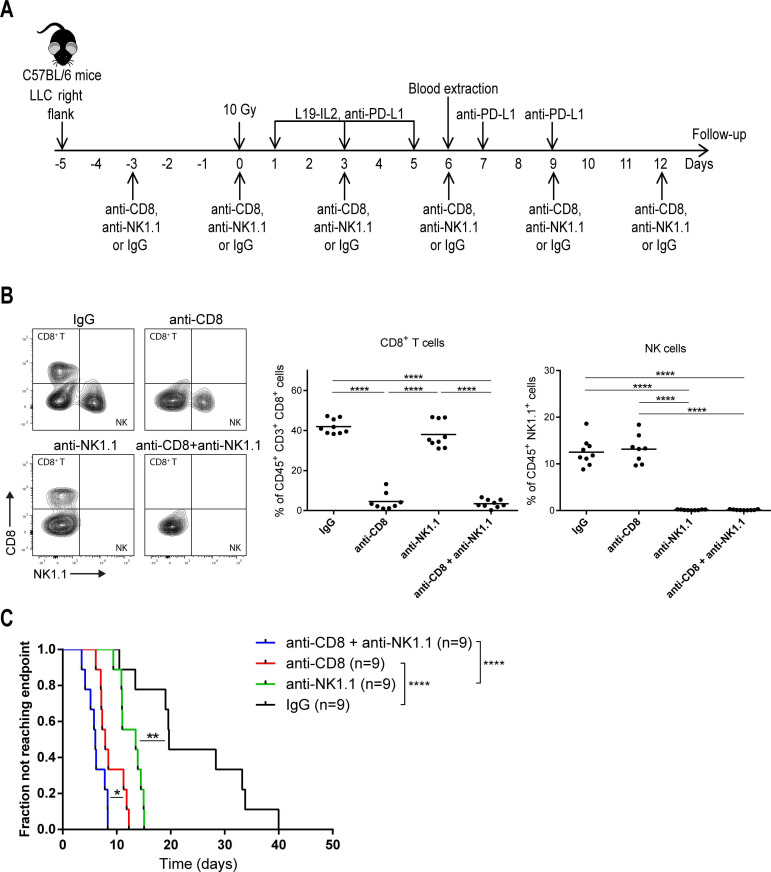
CD8^+^ T and NK cells play a key role in the anti-tumor effect of triple therapy. (A) Treatment schedule of the depletion study. (B) Flow cytometric analysis of CD8^+^ T cell and NK cell presence in the blood to confirm their depletion after 3 doses of depleting antibodies. (C) Fraction of tumors not reaching four times start tumor volume in LLC tumor-bearing mice (n=9 mice per treatment arm) treated with 10 Gy+L19–IL2+anti-PD-L1 depleted of CD8^+^ T (red), NK (green) or both cell types (blue) or IgG-treated (black) according to schedule in (A). This depletion study was performed once. IgG, immunoglobulin G; LLC, Lewis lung carcinoma.

To confirm the causal involvement of effector CD8^+^ T and NK cells in the anti-tumor effect triggered by the triple combination therapy, in vivo depletion of CD8^+^, NK1.1^+^ or both cell populations was performed in LLC-tumor-bearing mice (figure 4A). CD8^+^ T and NK cell depletion at day 6 after treatment start was confirmed in peripheral blood (2.4%±2.3% and 0.2%±0.1, respectively), as compared with IgG-treated mice (41.9%±3.6% and 12.5%±2.9, respectively; p<0.0001) (figure 4B). CD8^+^ T (p<0.0001) and NK (p<0.01) cell depletion reduced the therapeutic efficacy compared with the triple modality IgG-treated mice. Depletion of both CD8^+^ T and NK cells simultaneously further reduced therapeutic efficacy as compared with CD8^+^ T (p<0.05) or NK (p<0.0001) cell-depleted groups (figure 4C), demonstrating a causal role of both immune cell subsets in the RT+L19–IL2+anti-PD-L1 anti-tumor effect, with a major role by CD8^+^ T cells (p<0.01).

### Anti-PD-L1 treatment upregulates the expression of other IC molecules

**Figure 5:**
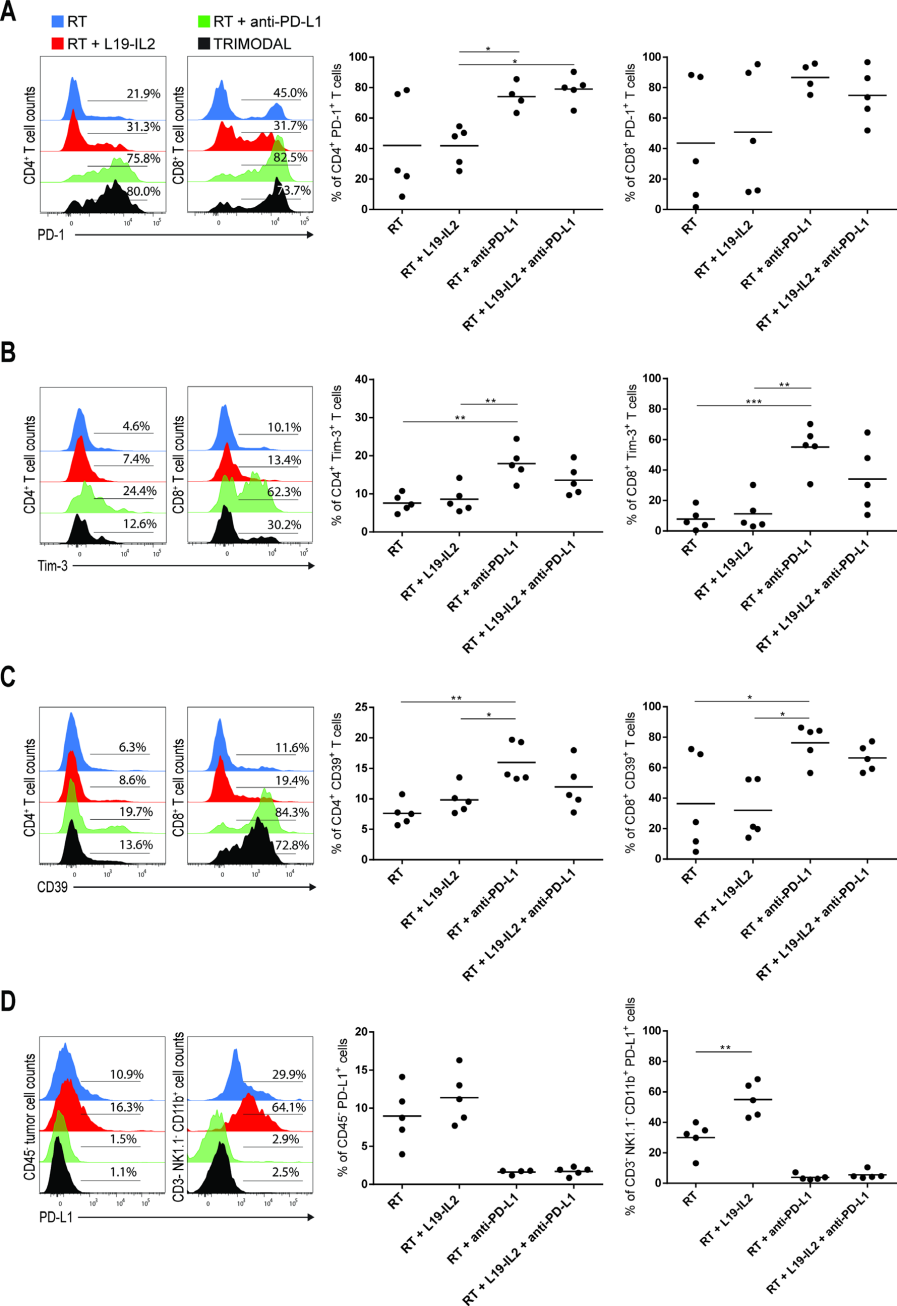
PD-L1 blockade upregulates the expression of other co-inhibitory receptors on tumor-infiltrating T cells. Representative flow cytometry histograms and quantifications of PD-1^+^ (A), Tim-3^+^ (B) and CD39^+^ (C) tumor-infiltrating CD4^+^ and CD8^+^ T cells from LLC tumors at day 6 after the start of treatment depicted in figure 2A. (D) Representative flow cytometry histograms of CD45− PD-L1^+^ LLC tumor cells and CD3− NK1.1− CD11b^+^ LLC tumor-infiltrating cells and quantifications. Experiment was performed once. Stainings, flow cytometry data acquisition and analysis of the samples were done in independent duplicates. LLC, Lewis lung carcinoma; RT, radiotherapy.

To further elucidate the underlying mechanisms of treatment response and resistance, we investigated immunosuppression within the tumor microenvironment (TME) by assessing expression of PD-1, Tim-3 and CD39 on tumor-infiltrating CD4^+^ and CD8^+^ T cells. High variability in percentages of CD4^+^ and CD8^+^ T cells expressing PD-1 was observed in RT-treated and RT+L19–IL2-treated tumors, while treatment with anti-PD-L1 led to the highest percent of T cells expressing PD-1 (figure 5A). Infiltration of CD8^+^ T cells expressing Tim-3 was low in tumors treated with RT only (7.8%±3.1) and RT+L19–IL2 (11.3%±5.1), but increased in tumors treated with RT+anti-PD-L1 (54.9%±8.5) and RT +L19–IL2+anti-PD-L1 (34.1%±9.9) (figure 5B). PD-L1 blockade also increased the expression of CD39, an ectoenzyme that hydrolyzes ATP into ADP causing immunosuppression on CD8^+^ T cells.^26^ Similar results were found for Tim-3 and CD39 expression on CD4^+^ T cells (figure 5C). Furthermore, the percentage of CD45− tumor cells expressing PD-L1 was not significantly altered between RT-treated and RT+L19–IL2-treated tumors and almost undetectable upon anti-PD-L1 treatment due to the therapeutic anti-PD-L1 antibody blocking the binding of the fluorescent detection antibody. This absence of detection is not equivalent to a lack of expression of PD-L1 on these cells, in fact, the success of this checkpoint inhibition therapy depends on the expression of PD-L1 on these (and other) cells. There were, however, increased proportions of tumor-infiltrating non-T, non-NK CD11b^+^ immune cells expressing PD-L1 (p<0.01) in LLC tumor-bearing mice treated with RT+L19–IL2 (55.0%±11.2), as compared with RT (30.0%±10.1) (figure 5D).

### RT+anti-PD-L1 increases tumor-infiltrating regulatory T cells

**Figure 6:**
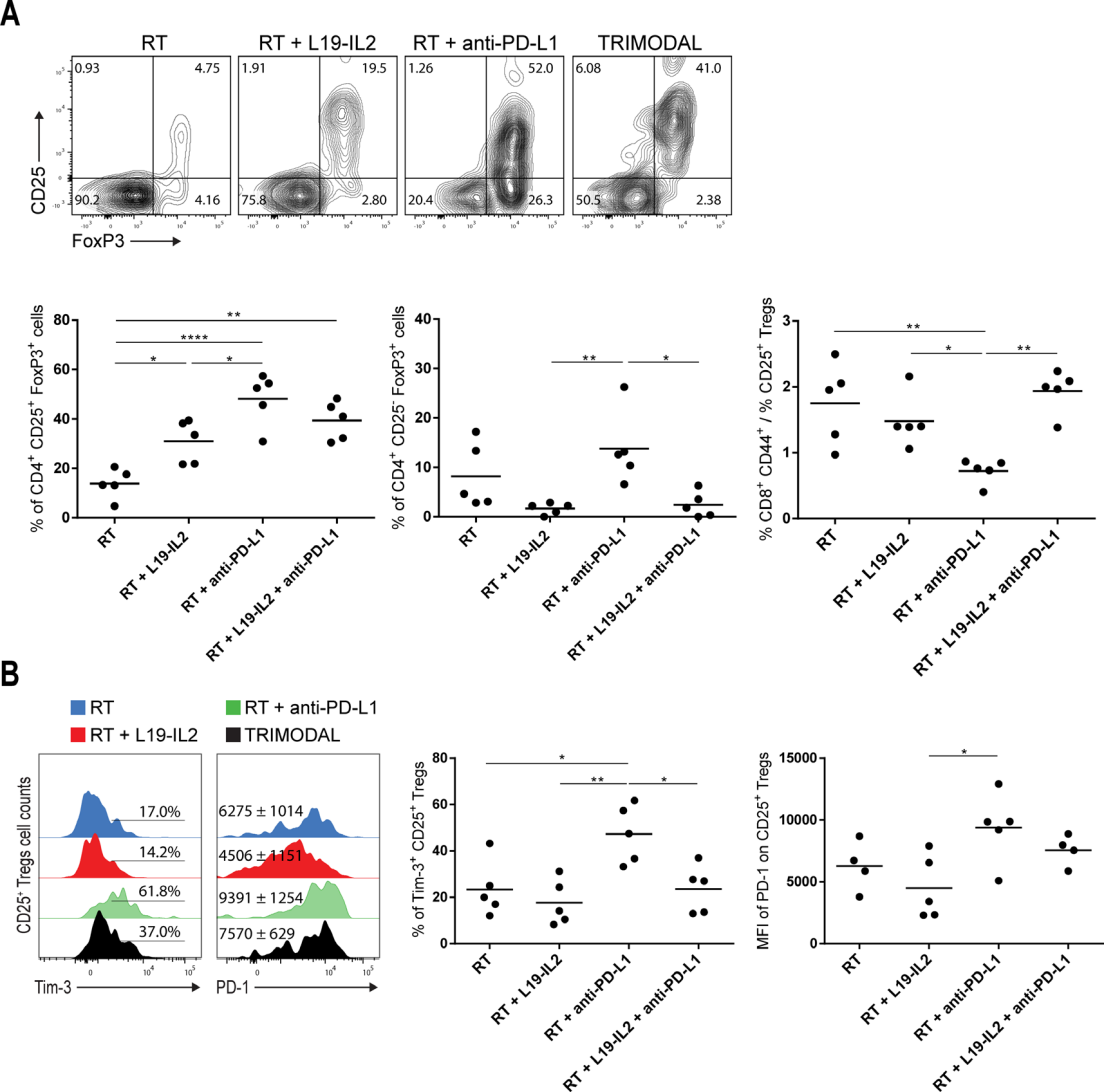
RT+anti-PD-L1 increases tumor-infiltrating Tregs. (A) Representative flow cytometry dot plots of tumor-infiltrating Tregs and quantification of CD4^+^ CD25^+^ FoxP3^+^ Tregs, CD4^+^ CD25^-^ FoxP3^+^ Tregs and CD8^+^ CD44^+^ T cell to CD25^+^ Treg ratio in LLC tumors at day six after the start of treatment depicted in figure 2A. (B) Representative flow cytometry histograms of PD-1^+^ and Tim-3^+^ tumor-infiltrating CD25^+^ Tregs and quantifications. Experiment was performed once. Stainings, flow cytometry data acquisition and analysis of the samples were done in independent duplicates. LLC, Lewis lung carcinoma; MFI, median fluorescence intensity; RT, radiotherapy; Tregs, regulatory T cells.

Next, we investigated the presence of regulatory T cells (Tregs) within tumors and whether they expressed co-inhibitory receptors 6 days after treatment onset. CD25^+^ Tregs were present in tumors from all treatment groups (figure 6A), with the lowest amount in RT-treated tumors (13.9%±2.7). L19–IL2 increased the proportions of CD25^+^ Tregs in tumors treated with RT+L19–IL2 (30.9%±3.9, p<0.05). Unexpectedly, the proportion of CD25^+^ Tregs was higher (p<0.05) in tumors treated with RT+anti-PD-L1 (48.2%±4.7) when compared with RT+L19–IL2-treated tumors (figure 6A, left). In the triple therapy group, the increase of tumor-infiltrating CD25^+^ Tregs is likely due to a combined effect of L19–IL2 and anti-PD-L1 compared with the RT group (39.4%±3.5, p<0.01). We also detected the presence of a CD25– Treg population in tumors treated with RT only (8.2%±2.9) and RT+anti-PD-L1 (13.8%±3.3). Also, CD25− Tregs were significantly increased in RT+anti-PD-L1 treated tumors when compared with RT+L19–IL2 (1.7%±0.5, p<0.01) and trimodal therapy (2.4%±1.2, p<0.05) treated tumors (figure 6A, middle). Additionally, the CD8^+^ CD44^+^/CD25^+^ Treg ratio in RT+anti-PD-L1 treated tumors was considerably lower compared with RT (p<0.05), RT+L19–IL2 (p<0.05) and triple therapy (p<0.01) (figure 6A, right).

In tumor-bearing mice treated with RT+anti-PD-L1, PD-1 expression was upregulated on CD25^+^ Tregs (median fluorescence intensity (MFI): 9391±1254) compared with RT+L19–IL2 (MFI: 4506±1151) (p<0.05). Likewise, proportions of CD25^+^ Tregs expressing Tim-3 from RT+anti-PD-L1 (47.3%±5.6) treated tumors were significantly higher compared with RT (23.5%±5.4, p<0.05), RT+L19–IL2 (17.7%±4.4, p<0.01), and triple therapy (23.7%±4.6, p<0.05) treated tumors (figure 6B).

### Triple combination therapy increases the proportion of tumor-specific memory CD8^+^ T cells in LLC-cured mice

**Figure 7:**
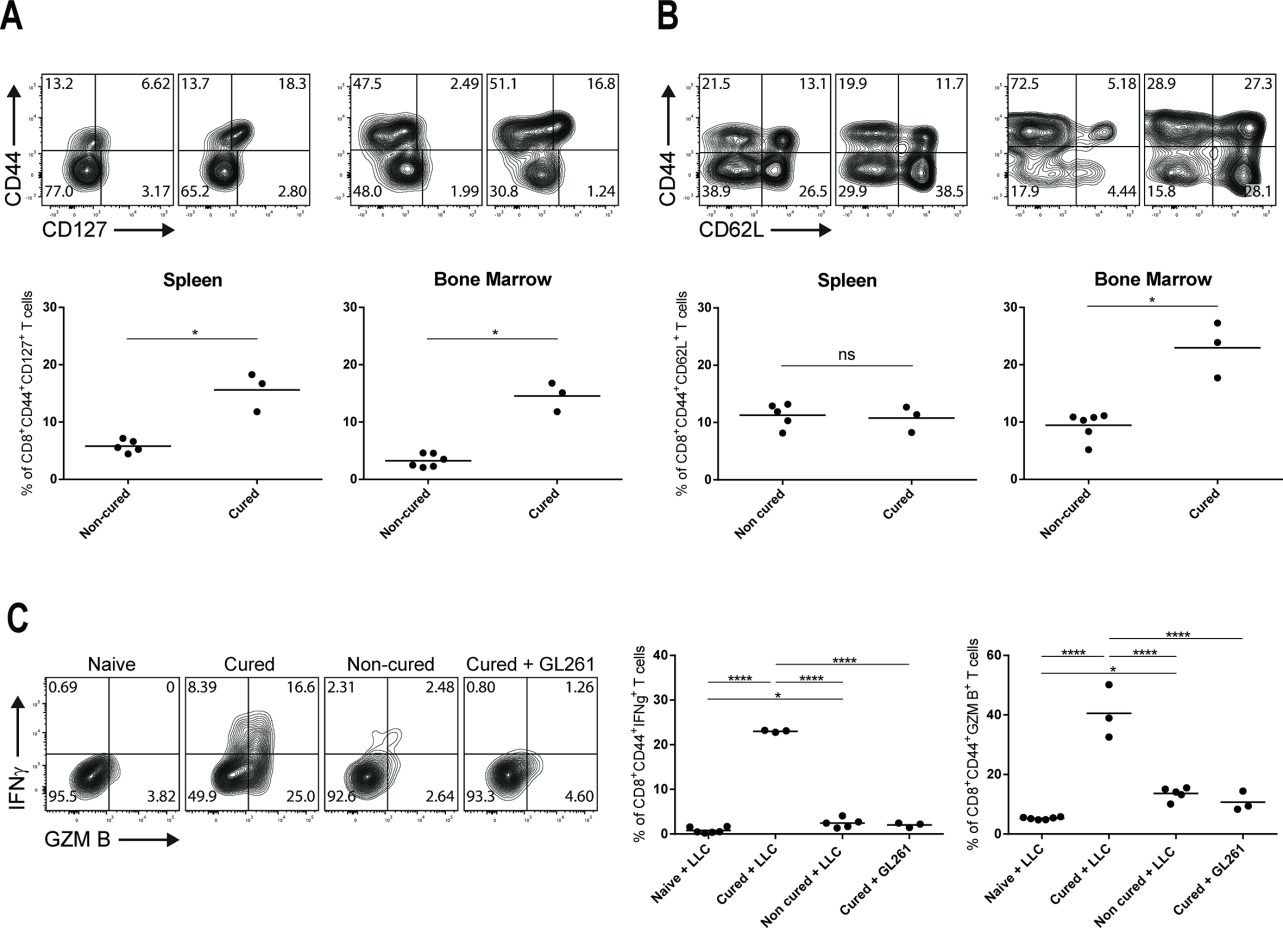
Anti-PD-L1-based trimodal therapy causes an accumulation of memory T cell subsets in lymphoid organs of cured LLC tumor-bearing mice. Representative flow cytometry dot plots and quantification of CD8^+^ CD44^+^ CD127^+^ memory T cells (A) and CD44^+^ CD62L^+^ central memory CD8^+^ T cells (B) from spleen and bone marrow of LLC tumor-bearing mice treated with 10 Gy+L19–IL2+anti-PD-L1 harvested 43±2.1 days after tumor cure (12 days after tumor rechallenge) or endpoint (T4×SV) had been reached. Cells were obtained from one experiment performed in figure 1B. Stainings, flow cytometry data acquisition, and analysis of the samples were done in independent duplicates. (C) Representative flow cytometry dot plots and quantifications of CD8^+^ CD44^+^ T cells expressing IFNγ and granzyme B from splenocytes of cured or non-cured LLC tumor-bearing mice treated with 10 Gy+L19–IL2+anti-PD-L1, or from naïve mice after co-culture with irradiated LLC target cells or GL261 as non-specific target cells. Cells were obtained from the same experiment as in (A) and (B). Co-culture assay, stainings, flow cytometry data acquisition, and analysis of the samples were done in independent duplicates. LLC, Lewis lung carcinoma.

To demonstrate that LLC-tumor bearing mice cured on RT+L19–IL2+anti-PD-L1 treatment developed a protective immune memory effect, we assessed the presence of central and CD127^+^ memory CD8^+^ T cells in the spleen and bone marrow of these mice. The CD8^+^ CD44^+^ CD127^+^ memory T cell subset was increased (p<0.05) in spleen (15.6%±1.9) and bone marrow (14.5%±1.4) of cured mice compared with non-cured mice (5.8%±0.4% and 3.2%±0.4, respectively) (figure 7A). Central memory CD8^+^ CD44^+^ CD62L^+^ T cell proportions were higher (p<0.05) in bone marrow (22.9%±2.8) of cured mice compared with non-cured mice (9.4%±0.9), but not in spleen (figure 7B). To assess the activity of CD8^+^ CD44^+^ T cells in the anti-tumor immune memory effect, splenocytes from cured, non-cured or naïve mice were co-cultured in vitro with LLC cells and the expression of IFNγ and granzyme B was evaluated after co-culture. Splenocytes of cured mice were also co-cultured with GL261 cells to prove antigen specificity. Splenocytes from cured mice had a higher (p<0.0001) proportion of CD8^+^ CD44^+^ T cells producing IFNγ (23.0%±0.1) or granzyme B (40.5%±5.1) compared with cells from non-cured (2.4%±0.4% and 13.6%±0.9, respectively) or naïve mice (0.8%±0.2% and 5.2%±0.1, respectively). Interestingly, 17.2%±1.4 of CD8^+^ CD44^+^ T cells produced both effector molecules. Co-cultures of splenocytes from cured mice with GL261 cells did not result in the production of IFNγ and granzyme B (figure 7C).

## Discussion

Poorly immunogenic tumors are notoriously difficult to cure and several previous attempts using combinatorial strategies with immunotherapies failed to do so.^27 28^ Here, we demonstrate for the first time that the combination of RT, L19–IL2 and anti-PD-L1 resulted in superior outcome in the immunogenically poor LLC model with a cure rate of 38% as compared with any bimodal treatment, which did not provide curative effects. On rechallenge of the cured animals, all remained tumor free. In agreement with previous data,^19^ we demonstrated that 10 Gy+L19–IL2+anti-PD-L1 can induce central and CD127^+^ memory T cells in LLC tumor-bearing mice showing complete remission. Since we performed the staining in cryopreserved cells, we acknowledge the possibility of CD62L cleavage due to cryopreservation. However, in our previous work, we performed the same stainings in fresh and cryopreserved cells and observed similar results,^19^ thus this possibility is unlikely. The synergistic effects between RT and immunotherapy is a topic of active investigation, especially because it has the potential to make cold tumors susceptible to immune attack.^29 30^ Herein, we studied the therapeutic outcome of combining single-dose RT with two types of immunotherapy (L19–IL2 and ICB) in the poorly immunogenic LLC tumor model^22 23^ and the T cell inflamed C51 and CT26 colon carcinoma models.^20 22 23^ The rationale of combining these three types of therapy relies on a “push the accelerator and release the brakes” approach, that is, combining (immuno)therapies that stimulate the immune system, in this case, RT and L19–IL2, with an immunotherapy that inhibits T cell exhaustion, such as ICB. In contrast to LLC, we found that C51 and CT26 tumors did not benefit from trimodal treatment. Responses yielded by RT+L19–IL2 and RT+L19–IL2+anti-CTLA-4 were comparable between the three tumor models. However, triple therapy with anti-PD-L1 was only efficacious in the LLC model. We found that this effect was associated with higher proportions of tumor-infiltrating NK, NKT and CD8^+^ T cells, and later on, confirmed dependency of the effect on NK and CD8^+^ T cells. Moreover, the analysis of immunological blood parameters across three different models indicates that triple therapy decreased PD-L1 expression most efficiently in LLC as compared with C51 and CT26 models in addition to the upregulation of PD-1 checkpoint molecule in the latter two tumor models, representing the resistance mechanism to the triple therapy. In addition, the most pronounced decrease in PD-L1 expression was found in the LLC model, suggesting that a significant response to the triple therapy may be partially due to more a efficient PD-L1 blockade in the LLC model. The differences in response to triple therapy might also be explained at least in part by differences in intrinsic tumor cell factors such as tumor mutational burden impacting the regulation on the kinetics of IC molecule expression between tumor models, that influences ICB efficacy, which requires further investigation. Our results suggest that the upregulation of checkpoint molecules in peripheral T cells induced by treatment might be responsible for resistance to trimodal therapy. It also appears that immunological parameters on peripheral T cells reflect the processes ongoing in the tumor, making it very attractive to develop biomarkers of tumor response.

Additionally, we observed that the use of L19–IL2 in combination with RT outperformed RT combined with ICB in the C51 model, while no differences were found between bimodal combinations in the CT26 model. Hot tumors are by definition tumors that are already relatively highly infiltrated by lymphocytes at baseline (that is, prior to any therapeutic intervention). According to literature and to our own findings, CT26 and C51 tumors are considered hot tumors since they have lymphocyte tumor infiltration at baseline^20 22 23^; however, our data indicate that C51 and CT26 tumors are resistant to ICB. It has been shown that hot tumors are a prerequisite to ICB response, however, not all hot tumors respond to ICB.^31^ For instance, ovarian cancer is considered a hot tumor and is resistant to ICB.^32 33^ Moreover, the majority of patients with melanoma and non-small cell lung cancer do not respond to ICB despite being classified as hot tumors.^34 35^ Other criteria intrinsic to the tumor cells themselves are arising as better predictors of ICB response, like, for example, tumor mutational burden. We believe that factors inherent to the tumor cells themselves could influence the differential response observed between these tumor models, regardless of the lymphocyte tumor-infiltration status and this is an interesting concept that requires further investigation.

Despite targeting the same pathway, combination therapy with anti-PD-L1 was more efficacious than anti-PD-1 in the LLC model. Although blockade of PD-1 and PD-L1 has been used interchangeably in cancer immunotherapy as a strategy to activate T cells, recent studies are showing that there are differential effects between anti-PD-1 and anti-PD-L1. PD-1 expression and signaling are exclusively on lymphocytes; on the other hand, PD-L1 can act as a receptor on tumor cells, lymphocytes and macrophages, transmitting a biochemical signal back into these cells on engagement with PD-1. This reverse signaling has different biological effects: anti-apoptotic on tumor cells,^36^ tumor-promoting tolerance on T cells^37^ and immunosuppressive on macrophages.^38^ Thus, while anti-PD-L1 can have effects on three different cell types involved in tumor immunity, anti-PD-1 acts only on lymphocytes. A recent study has shown that anti-PD-L1 blockade induced inflammatory signatures on both monocytes and T cells, in contrast to anti-PD-1 that induced different changes on gene expression predominately on T cells.^39^ Another study showed that anti-PD-L1 has a superior blocking capacity as compared with anti-PD-1 in vitro.^40^ The efficacy between antibodies could be also linked to the antibody isotype, since some isotypes have a longer half-life than others and some isotypes exhibit antibody-dependent cell cytotoxicity capacity which can influence the therapeutic effect. Another explanation for this differential response could be due to the fact that PD-L1 also binds to CD80. Anti-PD-L1 blocks the binding of these two molecules which could potentially augment T cell responses by blocking this pathway.^41^

We demonstrated that CD8^+^ T cells are involved in the anti-tumor response triggered by the trimodal treatment in LLC tumor-bearing mice. Although no differences were found in CD8^+^ T cell proportions in LLC tumors at day six after treatment onset between treatment arms, the activation status of these cells was higher in the trimodal-treated group, as determined by the expression of CD44, a marker for antigen-experienced and active T cells.^42^ Even if the correlation between CD8^+^ T cell tumor infiltration and increased survival across different tumor types is well established,^43–46^ other parameters such as the activation status, clonotype, subset and location^47^ of tumor-infiltrating effector T cells matter in terms of therapeutic outcome. Additionally, higher proportions of NKT cells in L19–IL2-treated LLC tumors and of NK cells in trimodal-treated LLC tumors were found. The role of NK and NKT cells in anti-tumor immune surveillance is well established in various tumor types.^48–50^ The depletion of NK1.1^+^ cells also abrogated the anti-tumor effect of trimodal treatment, confirming their involvement. Since both NK and NKT cells express the marker NK1.1, we cannot exclude that both subsets could have a different role in the observed immune-mediated tumor rejection after triple therapy delivery. This corroborates previous data on increased NK cell anti-tumor cytotoxicity following IL-2 administration^51^ and NK cell contribution to ICB response^52 53^ since they also express immunosuppressive IC molecules.^54–56^ As compared with the tumor growth delay experiments, in the depletion study the IgG-treated control group did not demonstrate tumor cure but significant growth delay. One possible explanation is that FcRs expressed by NK cells or other immune cell subsets are binding to the Fc region of the administered IgG antibodies and blocking the FcR functions, thus interfering with antibody-dependent cell cytotoxicity (ADCC), one of the main NK cell functions. A study using activating FcR-deficient mice demonstrated that the anti-tumor activity of the anti-PD-L1 antibody clone 10F.9G2, the same used in this study, is partly dependent on FcR-mediated ADCC.^57^ The same study showed that the rat IgG2b isotype antibody, the same isotype of the anti-PD-L1 and the IgG used in this study, can interact with all mouse FcR,^57^ supporting our hypothesis. Besides, it was recently reported that a humanized anti-PD-L1 antibody (Avelumab) was able to engage CD16, an Fc receptor, on NK cells and induce tumor cell killing via ADCC.^58 59^ These findings highlight the influence of the antibody isotype in ICB efficacy.

We demonstrated that PD-L1 blockade upregulated the expression of co-inhibitory molecules such as PD-1, Tim-3 and CD39 on tumor-infiltrating T cells, potentially limiting the efficacy of trimodal treatment and corroborating with previous studies showing that ICB upregulates the expression of co-inhibitory receptors, which participate in acquired resistance to ICB.^2 60 61^ Indeed, clinical studies have demonstrated better therapeutic effects when dual checkpoint blockade is used, although more toxic.^62 63^ Although PD-L1 expression on tumor cells as a predictive biomarker for response to anti-PD-L1 has limitations,^64^ new studies showed that host expression of PD-L1 on antigen-presenting cells is crucial for an effective response to anti-PD-L1.^65 66^ Therefore, we investigated the expression of PD-L1 on non-T non-NK CD11b^+^ tumor-infiltrating immune cells and found that PD-L1 was upregulated on these immune cells in RT +L19–IL2-treated tumors, further supporting the rationale of adding anti-PD-L1 to RT plus L19–IL2.

We observed the expected increase of tumor-infiltrating CD25^+^ Tregs in mice treated with L19–IL2. Remarkably, we found that RT+anti-PD-L1 increased CD25^+^ Tregs tumor infiltration to even higher proportions compared with L19–IL2 treated groups, since IL2 promotes the proliferation of CD25**^+^** Tregs, as CD25 (the high-affinity chain of the IL-2 receptor) is highly expressed by CD25^+^ Tregs.^67^ We also noticed the presence of a tumor-infiltrating CD25– Treg population in RT and RT+anti-PD-L1-treated groups. CD25– Tregs are induced in the periphery (different from thymus-derived CD25^+^ Tregs) from highly plastic tumor-infiltrating CD4^+^ T cells by immunosuppressive signals present in the TME^68^ and are highly immune-suppressive.^69^ Furthermore, we assessed the CD8^+^ CD44^+^/nTreg cell ratio across different treatment arms and found an association between lower ratios and impaired treatment response in tumors treated with RT +anti-PD-L1. Interestingly, triple therapy increased CD8^+^ CD44^+^/Treg ratio by both augmenting tumor-infiltrating CD8^+^ CD44^+^ T cells and diminishing Tregs proportions. In agreement with our findings, the CD8^+^/Treg ratio has been described as a predictor of treatment resistance to immunotherapies.^70 71^

We found increased proportions of Tregs expressing PD-1 and Tim-3 in RT+anti-PD-L1-treated LLC tumors when compared with Tregs from the other treatment arms, likely being a major mechanism of immune suppression. Tregs can express and upregulate co-inhibitory receptors, making them more immune-suppressive.^72^ Tim-3^+^ Tregs have been also shown to be induced by 10 Gy+anti-PD-L1 in an orthotopic mouse tumor model of head and neck squamous cell carcinoma (HNSCC) and to be the cause of treatment resistance^73^ and described as highly immune-suppressive in patients with HNSCC.^74^

Studies in mice have found that anti-CTLA-4 has a Treg depleting effect via ADCC^75 76^ being antibody isotype-dependent. Although we found high levels of Tregs in RT+L19–IL2-treated tumors, adding anti-CTLA-4 did not improve therapeutic outcomes. L19–IL2 and anti-CTLA-4 have been shown to have a curative synergistic effect in CT26 tumors that is superior to L19–IL2 monotherapy.^77^ In the present study, we only observed a trend towards improvement when comparing RT+L19–IL2 vs RT +L19–IL2+anti-CTLA-4 in the CT26 model. One major difference between studies is the use of different anti-CTLA-4 antibody clones. Another explanation could be due to the use of anti-CTLA-4 antibodies with different isotypes. In fact, a further study showed a dramatic difference in efficacy from 90% to 0% cure rate of two anti-CTLA-4 antibodies with the same specificity (clone 9D9), but with distinct isotype (IgG2a vs IgG2b) in the CT26 model.^75^ In the present study, we used an anti-CTLA-4 clone 9D9 and IgG2b isotype, which could explain the modest results obtained in combination therapy. Importantly, authors showed that anti-CTLA-4 with IgG2b isotype only induced a slight reduction of intratumoral Tregs as compared with the intratumor Treg depletion induced by the IgG2a isotype.^75^ Thus, it is likely that anti-CTLA-4 isotype IgG2b in our study did not induce a substantial intratumoral Treg reduction, which may contribute to treatment resistance of RT combined with L19–IL2 and/or ICB. Another study showed IL-2 improved anti-CTLA-4 efficacy in mouse models of fibrosarcoma and colon carcinoma.^78^ In patients, however, the objective response rate of IL-2 and anti-CTLA-4 combination was not superior to single treatment, although some patients showed durable cancer regressions on combined treatment.^79^

In summary, LLC is a highly aggressive, poorly immunogenic tumor model unresponsive to most immunotherapies.^22^ By combining single-dose RT with two distinct immunotherapies with non-redundant mechanisms, the immunogenicity of LLC tumors was enhanced, with increased infiltration of activated NK and CD8^+^ T cells and decreased T cell exhaustion and tumor-infiltrating Tregs, achieving curative responses. The conclusions of this study are two-fold: (a) RT synergizes better with tumor targeted-IL-2 than with ICB to elicit anti-tumor responses, thus patients not responding to ICB might benefit from other types of immunotherapy, and (b) combining ICB with other immunomodulating agents, both systemic and local, holds a great promise of increasing response rate to immunotherapies by turning cold into hot tumors and inducing durable disease control. The promising results of this novel triple combination in a poorly immunogenic lung cancer model prompted us to modify the clinical protocol prior to the start of the currently ongoing international multicenter randomized clinical phase 2 trial ImmunoSABR (NCT03705403) in stage IV non-small cell lung cancer patients.
