# Supplementary material for: Releasing the brakes of tumor immunity with anti-PD-L1 and pushing its accelerator with L19–IL2 cures poorly immunogenic tumors when combined with radiotherapy
Source: J Immunother Cancer. 2021 Mar 9;9(3):e001764. doi: 10.1136/jitc-2020-001764 (PMC7944996; doi:10.1136/jitc-2020-001764)
Supplement: Supplementary data [file jitc-2020-001764supp001.pdf]

Supplementary Table 1.

Mean ± SD of treatment start tumor volumes.

Figure	Treatment group	Mean ± SD tumor volume at start of treatment (mm^3)	P values
1B	RT + PBS + IgG (LLC)	232.08 ± 94.12	0.5839
1B	RT + L19-IL2 + IgG (LLC)	195.43 ± 72.98	
1B	RT + PBS + anti-CTLA-4 (LLC)	211.30 ± 54.80	
1B	RT + L19-IL2 + anti-CTLA-4 (LLC)	245.18 ± 86.88	
1B	RT + PBS + anti-PD-L1 (LLC)	243.06 ± 55.87	
1B	RT + L19-IL2 + anti-PD-L1 (LLC)	201.68 ± 28.75	
1B	RT + PBS + anti-PD-1 (LLC)	208.58 ± 76.03	
1B	RT + L19-IL2 + anti-PD-1 (LLC)	261.81 ± 106.3	
1C	RT + PBS + IgG (CT26)	266.76 ± 29.58	0.2774
1C	RT + L19-IL2 + IgG (CT26)	244.37 ± 80.79	
1C	RT + PBS + anti-CTLA-4 (CT26)	229.08 ± 26.77	
1C	RT + L19-IL2 + anti-CTLA-4 (CT26)	236.94 ± 38.21	
1C	RT + PBS + anti-PD-L1 (CT26)	211.56 ± 31.08	
1C	RT + L19-IL2 + anti-PD-L1 (CT26)	219.72 ± 57.80	
1D	RT + PBS + IgG (C51)	227.25 ± 53.65	0.5018
1D	RT + L19-IL2 + IgG (C51)	197.27 ± 22.56	
1D	RT + PBS + anti-CTLA-4 (C51)	222.41 ± 36.10	
1D	RT + L19-IL2 + anti-CTLA-4 (C51)	200.31 ± 43.66	
1D	RT + PBS + anti-PD-L1 (C51)	195.33 ± 16.29	
1D	RT + L19-IL2 + anti-PD-L1 (C51)	206.01 ± 37.79	
2B	RT + PBS + IgG (LLC)	235.86 ± 50.82	0.4065
2B	RT + L19-IL2 + IgG (LLC)	190.84 ± 61.46	
2B	RT + PBS + anti-PD-L1 (LLC)	235.86 ± 50.82	
2B	RT + L19-IL2 + anti-PD-L1 (LLC)	235.36 ± 29.04	
3C	RT + L19-IL2 + anti-PD-L1 + IgG (LLC)	220.22 ± 24.45	0.4815
3C	RT + L19-IL2 + anti-PD-L1 + anti-CD8 (LLC)	188.86 ± 18.59	
3C	RT + L19-IL2 + anti-PD-L1 + anti-NK1.1 (LLC)	179.30 ± 30.91	
3C	RT + L19-IL2 + anti-PD-L1 + anti-CD8 + anti-NK1.1 (LLC)	189.30 ± 23.29	
SF 4A	RT + PBS + IgG (CT26)	249.80 ± 26.26	0.143
SF 4A	RT + L19-IL2 + IgG (CT26)	232.13 ± 36.77	
SF 4A	RT + L19-IL2 + anti-CTLA-4 (CT26)	217.19 ± 32.81	
SF 4A	RT + L19-IL2 + anti-PD-L1 (CT26)	277.38 ± 60.76	
SF 4A	RT + PBS + anti-PD-1 (CT26)	249.25 ± 64.31	
SF 4A	RT + L19-IL2 + anti-PD-1 (CT26)	249.88 ± 55.70	
SF 4B	RT + PBS + IgG (C51)	261.21 ± 94.29	0.840
SF 4B	RT + L19-IL2 + IgG (C51)	233.15 ± 41.35	
SF 4B	RT + PBS + anti-PD-1 (C51)	277.50 ± 118.91	
SF 4B	RT + L19-IL2 + anti-PD-1 (C51)	243.94 ± 133.43	
