# Supplementary figures and images for: Releasing the brakes of tumor immunity with anti-PD-L1 and pushing its accelerator with L19–IL2 cures poorly immunogenic tumors when combined with radiotherapy
Source: J Immunother Cancer. 2021 Mar 9;9(3):e001764. doi: 10.1136/jitc-2020-001764 (PMC7944996; doi:10.1136/jitc-2020-001764)

## Supplementary Figure 4

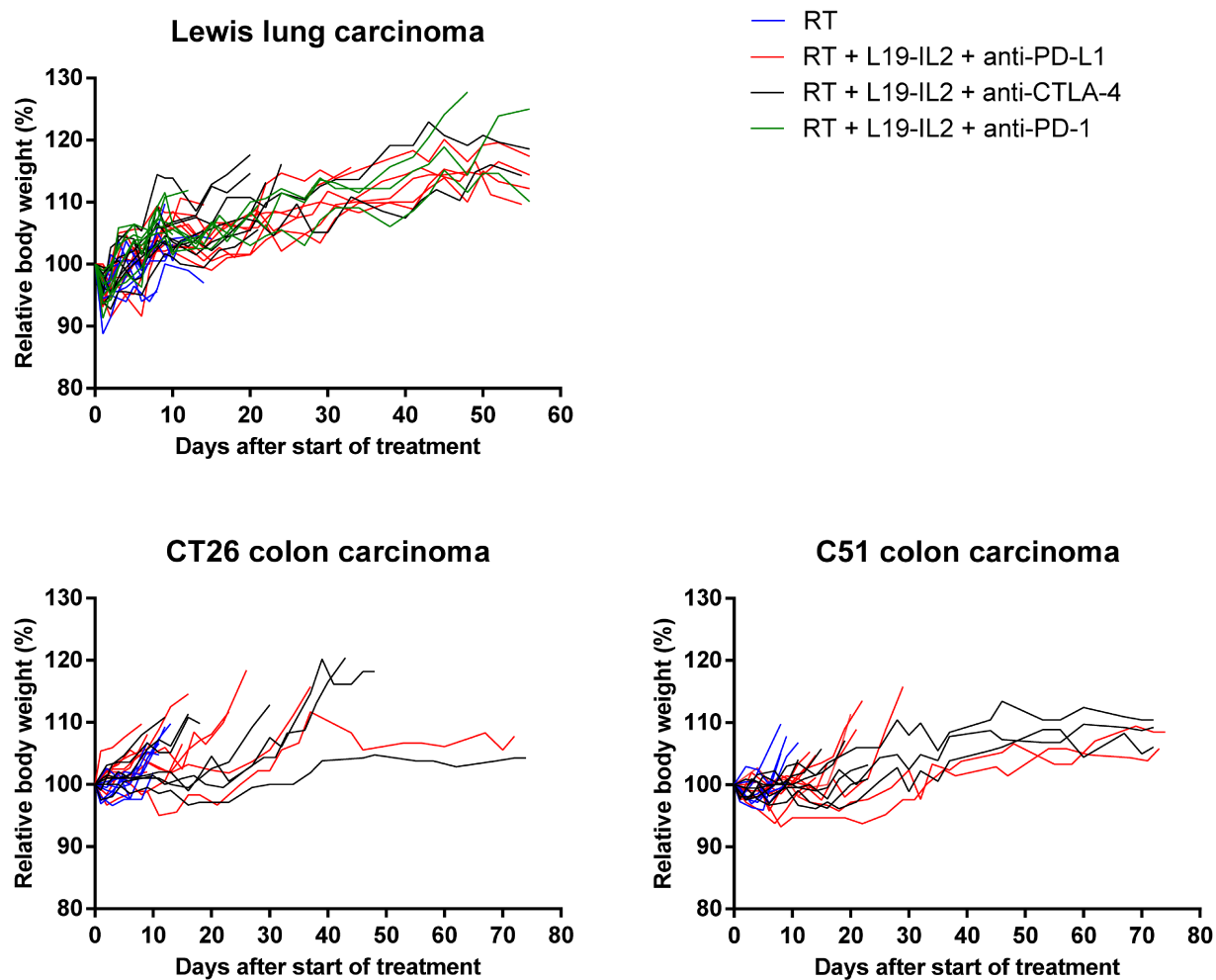

Supplement: Supplementary data [file jitc-2020-001764supp005.pdf]

## Supplementary Figure 1

A

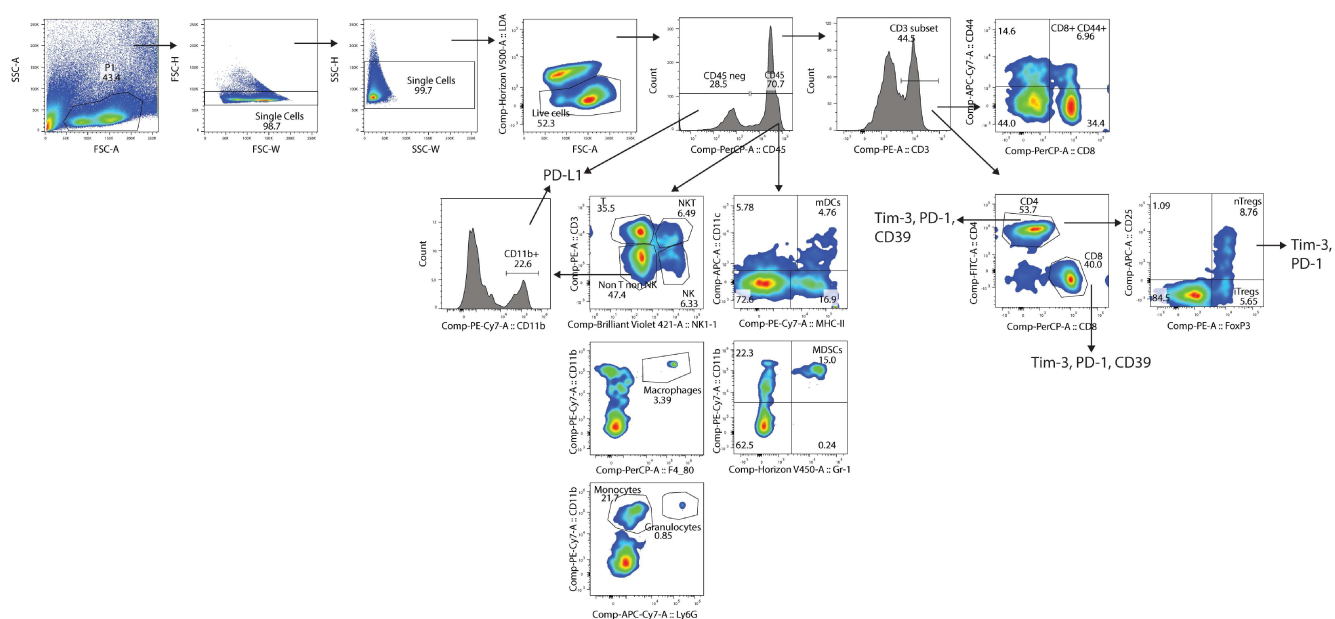

B

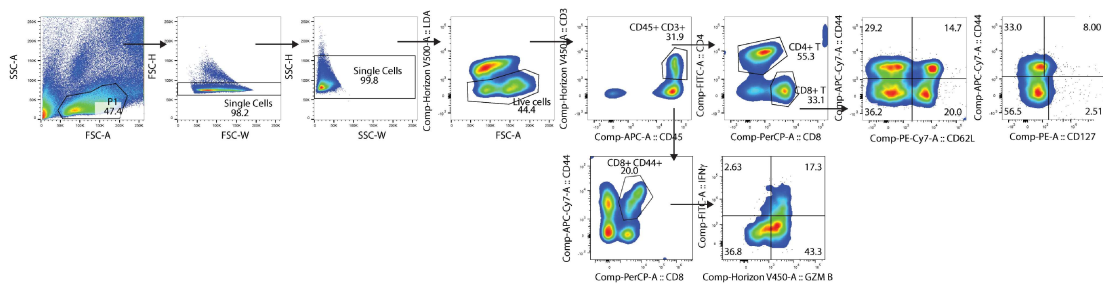

Supplement: Supplementary data [file jitc-2020-001764supp002.pdf]

## Supplementary Figure 3

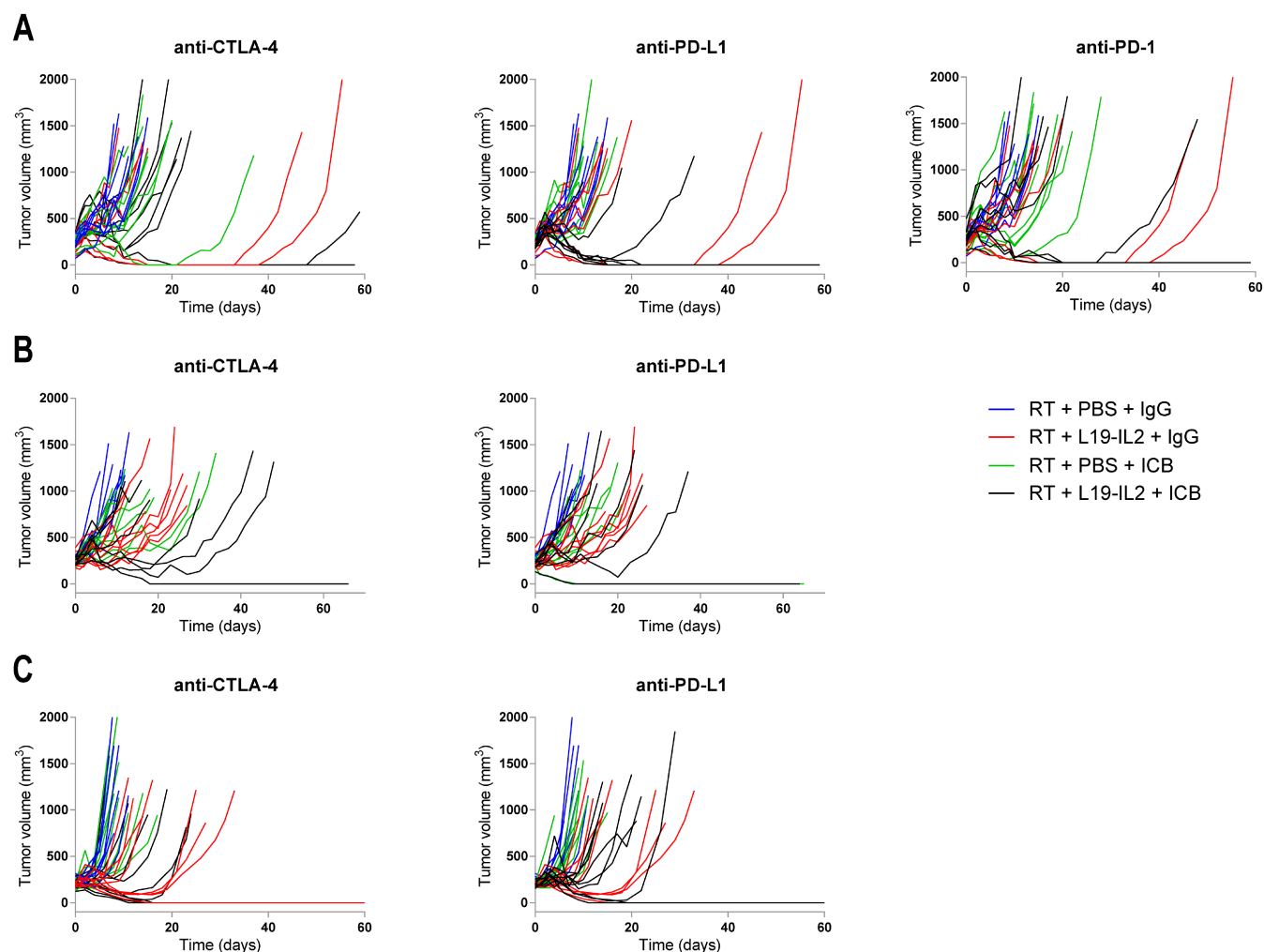

Supplement: Supplementary data [file jitc-2020-001764supp004.pdf]

## Supplementary Figure 5

A

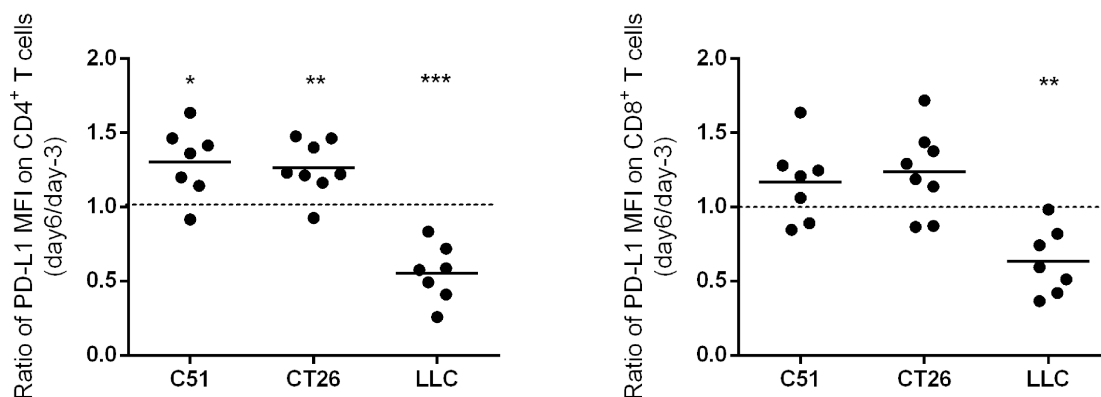

B

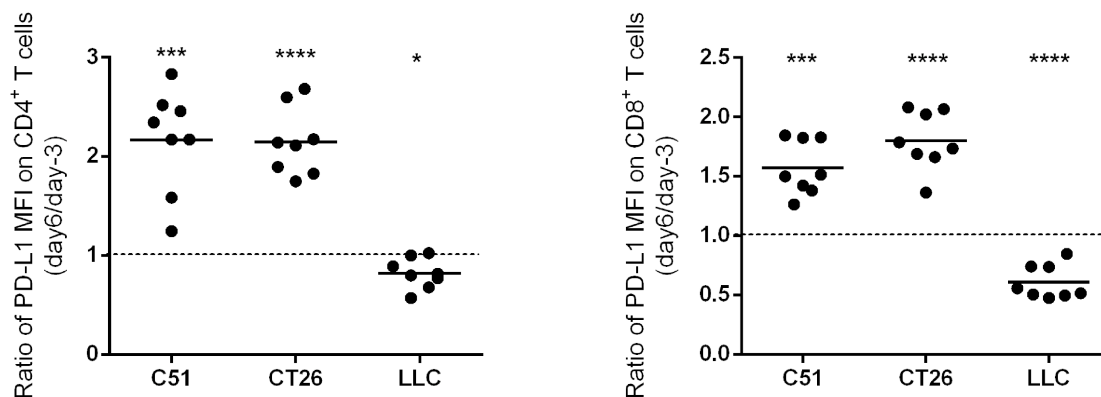

C

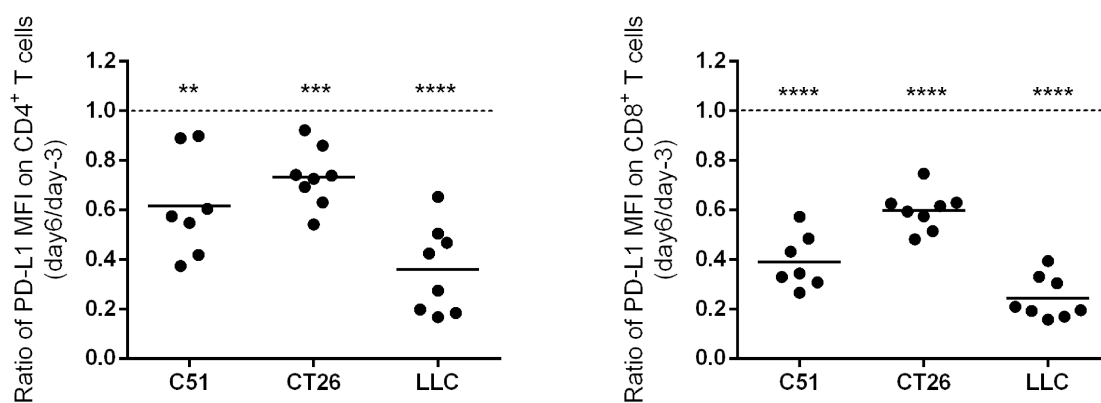

Supplement: Supplementary data [file jitc-2020-001764supp007.pdf]
