# Supplementary material for: Releasing the brakes of tumor immunity with anti-PD-L1 and pushing its accelerator with L19–IL2 cures poorly immunogenic tumors when combined with radiotherapy
Source: J Immunother Cancer. 2021 Mar 9;9(3):e001764. doi: 10.1136/jitc-2020-001764 (PMC7944996; doi:10.1136/jitc-2020-001764)
Supplement: Supplementary data [file jitc-2020-001764supp003.pdf]

Supplementary Figure 2

A

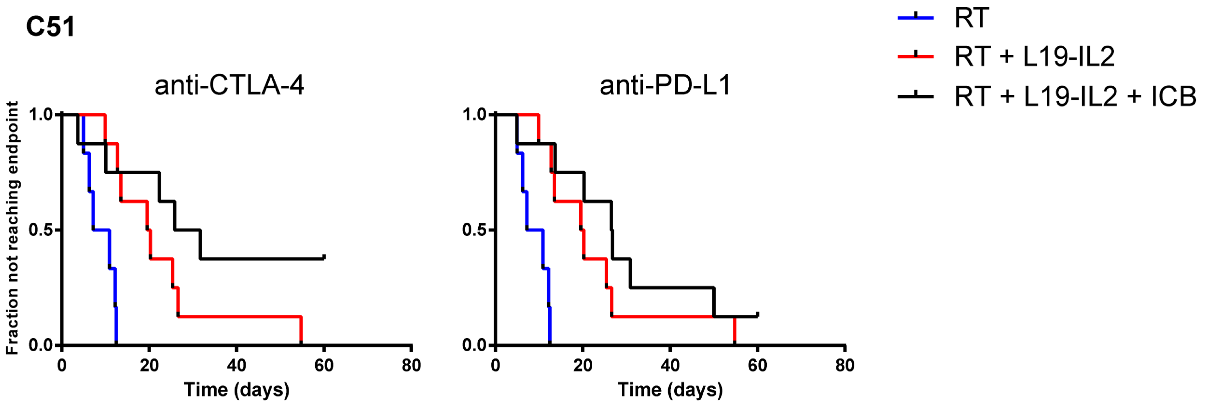

B

Tumor	Hazard ratio [95% CI] (p-value)		
	2-sided p-value vs. control, i.e. RT + L19-IL2		
	-	RT + L19-IL2 + anti-PD-L1	RT + L19-IL2 + anti-CTLA-4
**C51**	-	0.63 [0.22;1.75] (0.37)	0.41 [0.13;1.29] (0.13)
	*2-sided p-value vs. control, i.e. RT		
	RT + L19-IL2	RT + L19-IL2 + anti-PD-L1	RT + L19-IL2 + anti-CTLA-4
**C51**	**0.2 [0.05;0.68] (0.01)**	**0.12 [0.03;0.46] (0.002)**	**0.08 [0.02;0.34] (<0.0005)**

Significant HR and p values are shown in bold. *2-sided p-value vs. control, i.e. RT + L19-IL2. **2-sided p-value vs. control, i.e. RT.
