# Supplementary material for: Releasing the brakes of tumor immunity with anti-PD-L1 and pushing its accelerator with L19–IL2 cures poorly immunogenic tumors when combined with radiotherapy
Source: J Immunother Cancer. 2021 Mar 9;9(3):e001764. doi: 10.1136/jitc-2020-001764 (PMC7944996; doi:10.1136/jitc-2020-001764)
Supplement: Supplementary data [file jitc-2020-001764supp006.pdf]

**Supplementary Table 2.**

Mean percentages ± SD of tumor infiltrates from immune cell subsets from the myeloid line obtained by flow cytometric analysis at day 6 after treatment onset. MDSCs: myeloid-derived suppressive cells,

| | Monocytes ($Ly6G^-CD11b^+$) | MDSCs ($Gr1^+CD11b^+$) | Granulocytes ($Ly6G^+CD11b^+$) | Macrophages ($F4/80^+CD11b^+$) | mDCs ($MHC\text{-}II^+$ $CD11c^+$) |
|---|---|---|---|---|---|
| RT + PBS + IgG | 33.8% ± 12.9 | 2.9% ± 2.0 | 1.0% ± 0.3 | 2.1% ± 1.8 | 4.4% ± 1.8 |
| RT + L19-IL2 + PBS | 29.3% ± 8.4 | 3.3% ± 2.1 | 0.8% ± 0.4 | 1.3% ± 0.3 | 3.5% ± 2.2 |
| RT + PBS + anti-PD-L1 | 36.2% ± 11.3 | 3.1% ± 2.7 | 1.5% ± 0.3 | 1.6% ± 1.0 | 5.4% ± 2.5 |
| RT + L19-IL2 + anti-PD-L1 | 33.2 ± 10.2 | 4.3% ± 2.4 | 0.7% ± 0.3 | 1.5% ± 1.1 | 2.8% ± 1.4 |

mDCs: mature dendritic cells.
